# State-dependent associative plasticity highlights function-specific premotor-motor pathways crucial for arbitrary visuomotor mapping

**DOI:** 10.1126/sciadv.adu4098

**Published:** 2025-05-14

**Authors:** Sonia Turrini, Francesca Fiori, Giorgio Arcara, Vincenzo Romei, Giuseppe di Pellegrino, Alessio Avenanti

**Affiliations:** ^1^ Centro studi e ricerche in Neuroscienze Cognitive, Dipartimento di Psicologia “Renzo Canestrari,” Alma Mater Studiorum Università di Bologna Campus di Cesena, 47521 Cesena, Italy.; ^2^ NeXT: Neurophysiology and Neuroengineering of Human-Technology Interaction Research Unit, Campus Bio-Medico University, 00128 Rome, Italy.; ^3^ IRCCS San Camillo Hospital, Venice, Italy.; ^4^ Facultad de Lenguas y Educación, Universidad Antonio de Nebrija, Madrid 28015, Spain.; ^5^ Centro de Investigación en Neuropsicología y Neurociencias Cognitivas, Universidad Católica del Maule, Talca, Chile.

## Abstract

Arbitrary visuomotor mapping (AVMM) showcases the brain’s ability to link sensory inputs with actions. The ventral premotor cortex (PMv) is proposed as central to sensorimotor transformations, relaying descending motor commands through the primary motor cortex (M1). However, direct evidence of this pathway’s involvement in AVMM remains elusive. In four experiments, we used cortico-cortical paired associative stimulation (ccPAS) to enhance (ccPAS_PMv-M1_) or inhibit (ccPAS_M1-PMv_) PMv-to-M1 connectivity via Hebbian plasticity. Leveraging state-dependent properties of transcranial magnetic stimulation, we targeted function-specific visuomotor neurons within the pathway, testing their physiological/behavioral relevance to AVMM. State-dependent ccPAS_PMv-M1_, applied during motor responses to target visual cues, enhanced neurophysiological and behavioral indices of AVMM, while ccPAS_M1-PMv_ had an opposite influence, with the effects being more pronounced for target relative to control visual cues. These results highlight the plasticity and causal role of spatially overlapping but functionally specific neural populations within the PMv-M1 pathway in AVMM and suggest state-dependent ccPAS as a tool for targeted modulation of visuomotor pathways.

## Teaser

State-dependent neuroplasticity affects physiological and behavioral outputs of functionally specific premotor-motor projections.

## INTRODUCTION

Imagine learning to drive in a country with unfamiliar traffic signs. Each new sign requires you to learn and associate a visual cue with a specific motor action, like braking or turning, despite the lack of an inherent connection between them. This process, known as arbitrary visuomotor mapping (AVMM), is crucial not just for driving but for many everyday tasks—from using new technology to following instructions on unfamiliar devices. Understanding the neural mechanisms underlying AVMM is essential, as this ability forms the foundation of our capacity to adapt flexibly to novel environments and complex challenges (*1*). Following seminal findings about the role of the premotor cortex role in AVMM (*2*), which initially emphasized the dorsal sector (*3*), subsequent research has identified numerous areas involved in encoding visuomotor associations (*1*, *4*–*6*). The ventral premotor cortex (PMv) garnered particular interest because of its response patterns: Single-cell studies reveal that PMv neurons exhibit learning-related activity (*7*), rapidly attuning to specific sensory features like colors (*8*) and transforming sensory information into motor commands (*9*), with learned visuomotor associations maintained even after the end of the task, once they are no longer relevant (*8*). While this evidence suggests a role of PMv in AVMM, inconsistent results have been reported (*10*).

The PMv has been proposed as a key hub for standard, non-arbitrary visuomotor transformations associated with grasping objects with different shapes and sizes (*11*, *12*). Consistent with this role, PMv neurostimulation has been shown to affect sensory-guided grasping (*13*, *14*), although performance changes have also been observed in simple reaction tasks (*15*). The PMv conveys descending motor commands mainly through the primary motor cortex (M1) (*16*, *17*). This anatomical arrangement suggests a functional hierarchy where PMv processes and integrates sensory information to form visuomotor associations, which are then transmitted to M1 for execution. Consistent with this model, learning visuomotor tasks increases PMv-M1 functional coupling (*18*–*20*), highlighting the potential role of the PMv-M1 pathway in encoding and implementing visuomotor associations. However, direct evidence causally linking this specific pathway to AVMM has remained elusive.

To address this gap, we adopted a network-based transcranial magnetic stimulation (TMS) approach using cortico-cortical paired associative stimulation (ccPAS). In four experiments, we modulated the strength of the PMv-to-M1 pathway while healthy humans actively engaged in an AVMM task under various conditions. Inspired by the Hebbian plasticity principle, the ccPAS protocol involves repeated asynchronous paired stimulation of two cortical areas, mimicking neuronal activity patterns known to induce spike timing–dependent plasticity (STDP) (*21*–*25*). Previous evidence suggests that ccPAS can transiently modulate the strength of directional connectivity between targeted sites, leading to long-term potentiation (LTP)–like effects when the presynaptic area is stimulated before the postsynaptic area (i.e., ccPAS_PMv-M1_) and long-term depression (LTD)–like phenomena when the stimulation order is reversed (i.e., ccPAS_M1-PMv_), consistent with the principles of Hebbian STDP (*21*, *26*–*28*). While prior work has shown that the offline application of ccPAS over the PMv-to-M1 pathway can affect performance on tasks requiring its efficient communication (*29*, *30*), these approaches suffer from reduced spatial and functional specificity (*22*, *31*, *32*), as offline TMS applications are nonspecific with regard to the functional type of neurons they target within the stimulated area. To overcome this limitation, we integrated ccPAS with a concurrent AVMM task, hypothesizing that this would enhance the functional specificity of the stimulation (*22*, *33*). By applying ccPAS over PMv-M1 while activating a specific visuomotor association (i.e., a visual cue paired with a finger movement), we capitalize on the principle of state-dependent TMS effects, by which the neural state at the time of stimulation shapes the outcomes (*24*, *31*, *34*). Thus, using a state-dependent ccPAS approach—combining the manipulation of the neural state via the AVMM task and cortical stimulation—we aimed to selectively alter the synaptic efficiency of functionally specific visuomotor networks within the PMv-to-M1 pathway via STDP.

## RESULTS

### State-dependent ccPAS highlights function-specific premotor-motor networks during AVMM

**Fig. 1.**
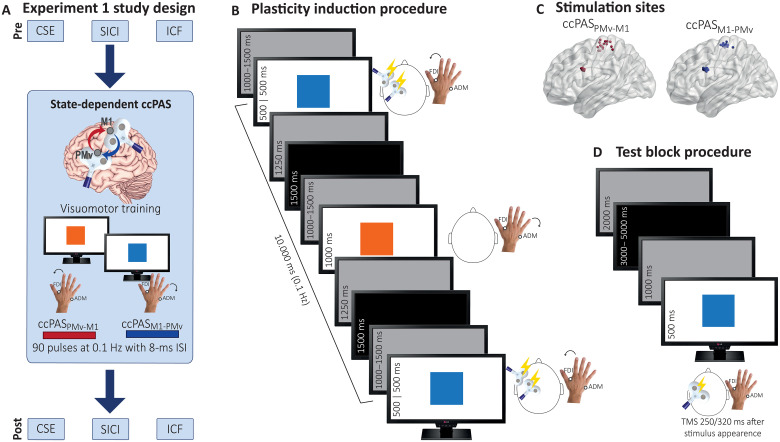
Experiment 1 methodology. (**A**) General design. We assessed CSE, SICI, and ICF in three separate test blocks before and after state-dependent ccPAS. In two different sessions, participants were submitted to ccPAS_PMv-M1_ and ccPAS_M1-PMv_ during an AVMM task. (**B**) Plasticity induction procedure. State-dependent ccPAS paired pulses were administered 500 ms after the onset of the target visual cue while participants were responding with a finger movement involving the target muscle (e.g., the FDI muscle in response to the blue square). During the control visual cue, requiring a movement involving the control muscle no TMS pulses were delivered. The two cues appeared alternatively, for a total of 90 trials for each color. (**C**) Individual targeted cortical sites reconstructed on a standard template using MRIcron software (MRIcron/NPM/dcm2nii) after conversion to MNI space for illustrative purposes. (**D**) Test block procedure. TMS pulses were administered over M1 to induce MEPs at 250 to 320 ms after the onset of the target and control visual cues and assess CSE, SICI, and ICF.

In experiment 1, we aimed to target functionally specific neural populations within the PMv-M1 pathway through the state-dependent application of ccPAS while participants engaged in AVMM. We tested the effect of state-dependent ccPAS on different indices of motor excitability (Fig. 1A). A group of 16 healthy young adults performed a simple AVMM task requiring the abduction of the right index finger [controlled by the first dorsal interosseous (FDI)] and the right little finger [controlled by the abductor digiti minimi (ADM)] in response to arbitrarily associated visual cues (an orange or blue square) presented on a computer screen (Fig. 1B). Participants performed the AVMM task in two sessions during which we applied two different ccPAS protocols over PMv and M1 in the left hemisphere (Fig. 1C). In the ccPAS_PMv-M1_ session, PMv stimulation was followed by M1 stimulation, with an 8-ms interstimulus interval (ISI) to activate and reinforce short-latency PMv-to-M1 connections (*35*–*39*), in line with the notion that coupling pre- and postsynaptic activity leads to LTP phenomena of STDP (*40*). In the ccPAS_M1-PMv_ session, the order of the two pulses was reversed, with the M1 pulse always delivered 8 ms before the PMv pulse to induce LTD-like effects within the PMv-M1 pathway (*41*). Although previous ccPAS studies have yielded mixed evidence regarding LTD, with some studies reporting its occurrence (*30*, *36*, *42*) and others failing to observe it (*29*, *35*, *43*, *44*), by using a brain-state coupled method, we sought to achieve a more consistent and reliable suppressive effect of the ccPAS_M1-PMv_ protocol.

In both sessions, every paired stimulation composing the ccPAS protocol was applied during each iteration of one specific visuomotor association of the AVMM task, i.e., the “target association,” entailing the contraction of the “target muscle” in response to the associated “target color” (e.g., the abduction of the index finger in response to the blue cue) (Fig. 1B). Only half of the visual cues and associated finger movements were coupled with TMS. The resulting state-dependent ccPAS protocol was composed of 90 pairs of TMS pulses delivered at a frequency of ~0.1 Hz (*35*, *36*, *45*). The cue-to-finger associations were counterbalanced across participants, and electromyographic (EMG) activity from the two muscles was recorded during the task, ensuring accurate AVMM performance (fig. S1). To test the effect of the two state-dependent ccPAS protocols, we recorded motor-evoked potentials (MEPs) induced by single-pulse TMS of the left M1 in the target and control muscles. In this way, we assessed corticospinal excitability (CSE) before and after the intervention. Critically, during CSE recording, participants were presented with the same visual cues viewed during the AVMM task, to assess their neurophysiological response to the same cues they learned to associate with a finger movement (Fig. 1D).

**Fig. 2.**
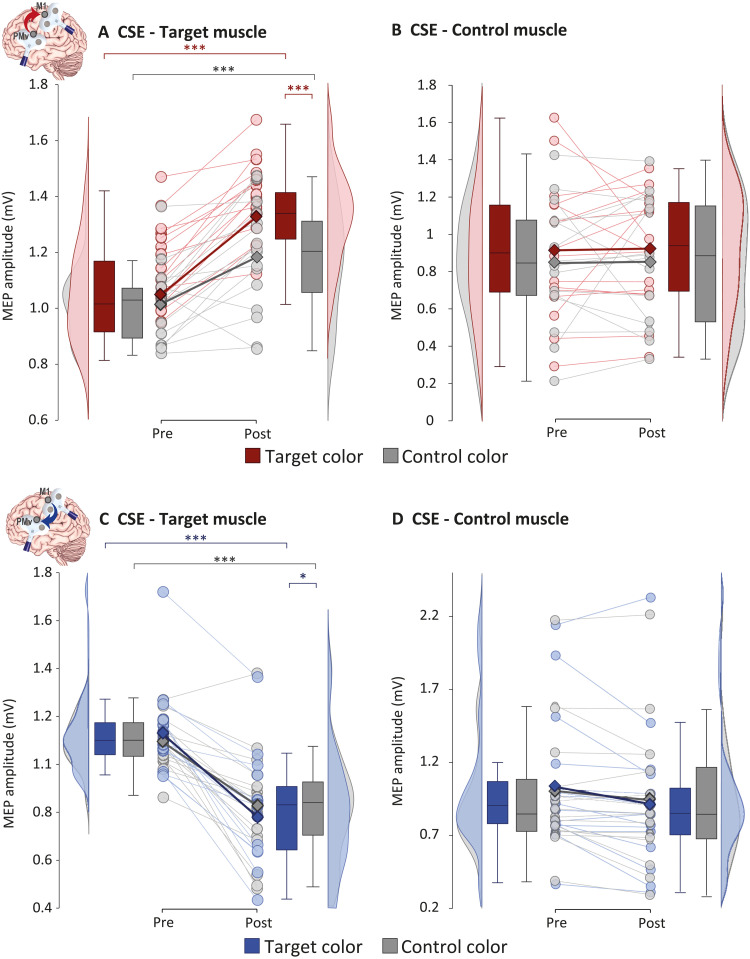
Results of experiment 1, testing the influence of state-dependent ccPAS. (**A**) Following ccPAS_PMv-M1_, CSE increased in the target muscle, and the facilitatory effect was more prominent for the target visual cue (red line). (**B**) No CSE change was observed in the control muscle. (**C**) After ccPAS_M1-PMv_, CSE decreased in the target muscle, with a more pronounced inhibitory response to target visual cues (blue line). (**D**) No CSE change was observed in the control muscle. Lighter circles and lines denote individual data points; darker squares and lines denote group means. Box plots represent the median, first and third quartiles, and the 95% confidence interval of individual data points. Asterisks indicate significant post hoc comparison: *P < 0.05 and ***P < 0.001.

In keeping with our hypotheses, the session (ccPAS_PMv-M1_ and ccPAS_M1-PMv_) × time (pre and post) × muscle (target and control) × color (target and control) analysis of variance (ANOVA) on MEP amplitudes revealed opposite modulations in CSE following the two ccPAS protocols, as shown by the higher-order interaction [*F*_1,15_ = 8.45; *P* = 0.011; partial η^2^ (η_p_^2^) = 0.360]. In the target muscle (Fig. 2A), whose activation was marked by ccPAS during performance of AVMM, we observed highly specific modulations of CSE (session × time × color interaction: *F*_1,15_ = 21.59; *P* < 0.001; η_p_^2^ = 0.590): CSE increased after state-dependent ccPAS_PMv-M1_ both when participants viewed the target color (Δ = 0.277 mV; *P* < 0.001; *d*_rm_ = 2.03) and the control one (Δ = 0.166 mV; *P* < 0.001; *d*_rm_ = 0.94), but, critically, this modulation was significantly larger for the target color (Δ = 0.144 mV; *P* < 0.001; *d*_rm_ = 1.28). In contrast, state-dependent ccPAS_M1-PMv_ had a strong suppressive effect (Fig. 2C): CSE decreased both when the target (Δ = −0.352 mV; *P* < 0.001; *d*_rm_ = 1.97) and the control color (Δ = −0.270 mV; *P* < 0.001; *d*_rm_ = 1.32) were presented, with a slight but significant difference between the two at post (Δ = −0.046 mV; *P* = 0.043; *d*_rm_ = 0.507) indicating greater suppression when viewing the target color. No consistent modulation of CSE was observed in the control muscle following either ccPAS intervention (all *F* ≤ 2.55, all *P* ≥ 0.13; Fig. 2, B and D).

In summary, state-dependent ccPAS, applied during motor responses to target visual cues, induced a bidirectional modulation of CSE that was dependent on the direction of the stimulation, with ccPAS_PMv-M1_ leading to increased CSE and ccPAS_M1-PMv_ leading to reduced CSE. These modulations were specific for the target muscle, showing motor selectivity. The modulations were also visuomotor specific, as both the excitatory effects of ccPAS_PMv-M1_ and the inhibitory effects of ccPAS_M1-PMv_ were more pronounced for target relative to control visual cues.

### Testing the specificity of function-specific premotor-motor networks during AVMM

**Fig. 3.**
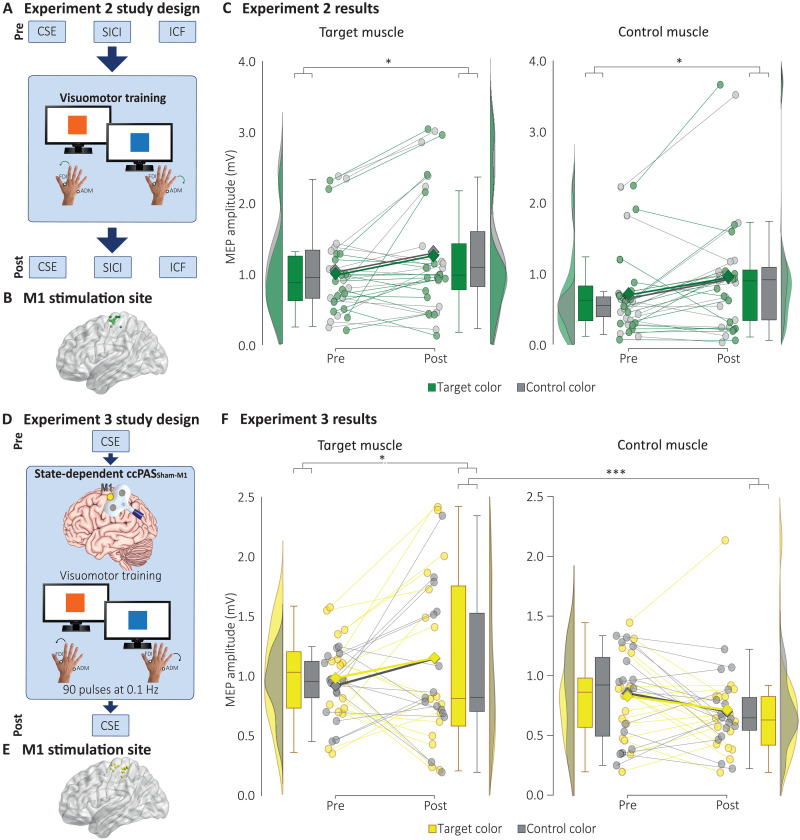
Methodology and results of experiments 2 and 3. (**A** to **C**) Experiment 2 testing the influence of AVMM without ccPAS. (A) Design. (B) Individual targeted sites reconstructed on a standard template using MRIcron software (MRIcron/NPM/dcm2nii) after conversion to MNI space for illustrative purposes. (C) Results: Following the AVMM task, CSE increased in both muscles and irrespective of the presented visual cue. (**D** to **F**) Experiment 3 testing the influence of state-dependent ccPAS_Sham-M1_. (D) Design. (E) Individual targeted cortical sites. (F) Results: State-dependent ccPAS_Sham-M1_ increased CSE in the target muscle only, irrespective of the presented visual stimulus. Lighter circles and lines denote individual data points; darker squares and lines denote group means. Box plots represent the median, first and third quartiles, and the 95% confidence interval of individual data points. *P < 0.05, and ***P < 0.001.

CSE findings in experiment 1 were specific to the state-dependent combination of ccPAS with AVMM. In experiment 2, performed on a new group of 16 healthy young adults, we investigated the CSE modulations induced by the same AVMM task used in experiment 1 but without the concurrent ccPAS applications (Fig. 3, A and B). The time × muscle × color ANOVA revealed a general increase in CSE in both muscles (Fig. 3C), irrespective of the presented visual cue, as shown by the main effect of time (*F*_1,15_ = 5.47; *P* = 0.034; η_p_^2^ = 0.267), which did not interact with any other factor (all *P* ≥ 0.50).

Repeated stimulation of M1 during motor performance can modulate CSE in the moving muscles (*46*). To specifically assess the effects of manipulating connectivity within the visuomotor stream (PMv-M1), rather than acting on its motor component alone (M1), we tested an additional group of 16 individuals in experiment 3. In this experiment, we combined the AVMM task with a modified ccPAS (ccPAS_Sham-M1_) involving active stimulation of M1 and sham stimulation of the PMv site, thus consisting of the repeated stimulation of M1 (Fig. 3, D and E). We hypothesized that the repeated stimulation of M1 during AVMM could enhance the motor—but not the visuomotor—selectivity of the training. That is, we expected that the stimulation of M1 during the execution of the target finger movements would enhance the CSE of the corresponding target muscle selectively, leaving the CSE of the control muscle unaltered (*46*). We expected this muscle specificity to occur without the stark visuomotor specificity observed in experiment 1 when comparing the target versus control color. Our findings support this hypothesis. The time × muscle × color ANOVA revealed a time × muscle interaction (*F*_1,15_ = 9.42; *P* = 0.008; η_p_^2^ = 0.386; Fig. 3F): While the CSE of the two muscles did not differ at baseline (*P* = 0.15), it increased in the target muscle (Δ = 0.199 mV; *P* = 0.021; *d*_rm_ = 0.35) and was significantly higher compared to the control muscle at post (Δ = 0.451 mV; *P* < 0.001; *d*_rm_ = 0.64). Critically, no effect of the presented visual cue was observed (all *P* ≥ 0.33).

**Fig. 4.**
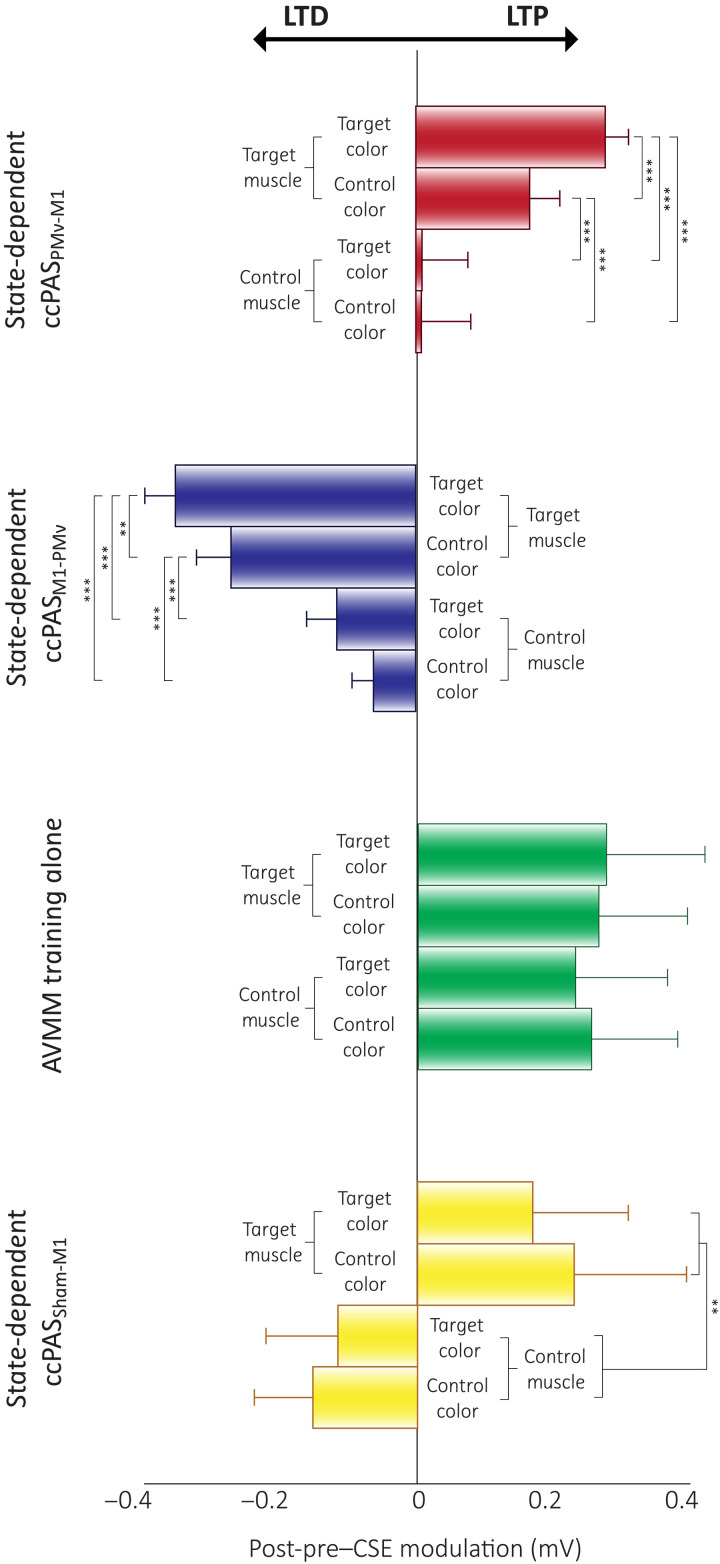
Summary of results of experiments 1 to 3. LTP-like enhancement of visuomotor specificity following state-dependent ccPAS_PMv-M1_ (red), LTD-like visuomotor-specific effect following state-dependent ccPAS_M1-PMv_ (blue), nonspecific CSE increase following the AVMM task with no TMS (green), and motor-specific LTP-like effect following state-dependent M1 stimulation (yellow). Histograms represent the mean of each condition; error bars represent 1 SEM. **P < 0.01 and ***P < 0.001.

Therefore, we conclude that the effects observed in experiment 1 cannot be attributed solely to the execution of the AVMM training, which, when performed without concurrent ccPAS, as in experiment 2, resulted in a general increase in CSE. Similarly, they cannot be explained by state-dependent stimulation of M1 alone, which produced a motor-selective but not a visuomotor-specific increase in CSE, as shown in experiment 3. A graphical representation of findings from experiments 1 to 3 can be found in Fig. 4.

### Changes in intracortical excitability induced by state-dependent ccPAS

To clarify the inhibitory and excitatory intracortical mechanisms underlying CSE modulations, in experiments 1 and 2, we also administer paired-pulse stimulation of M1 to assess short-interval intracortical inhibition (SICI) and intracortical facilitation (ICF) (*47*, *48*). For each participant, the analysis of SICI and ICF was restricted to the target muscle, for which stimulation parameters [i.e., resting motor threshold (rMT) and SI_1mV_] were optimized.

**Fig. 5.**
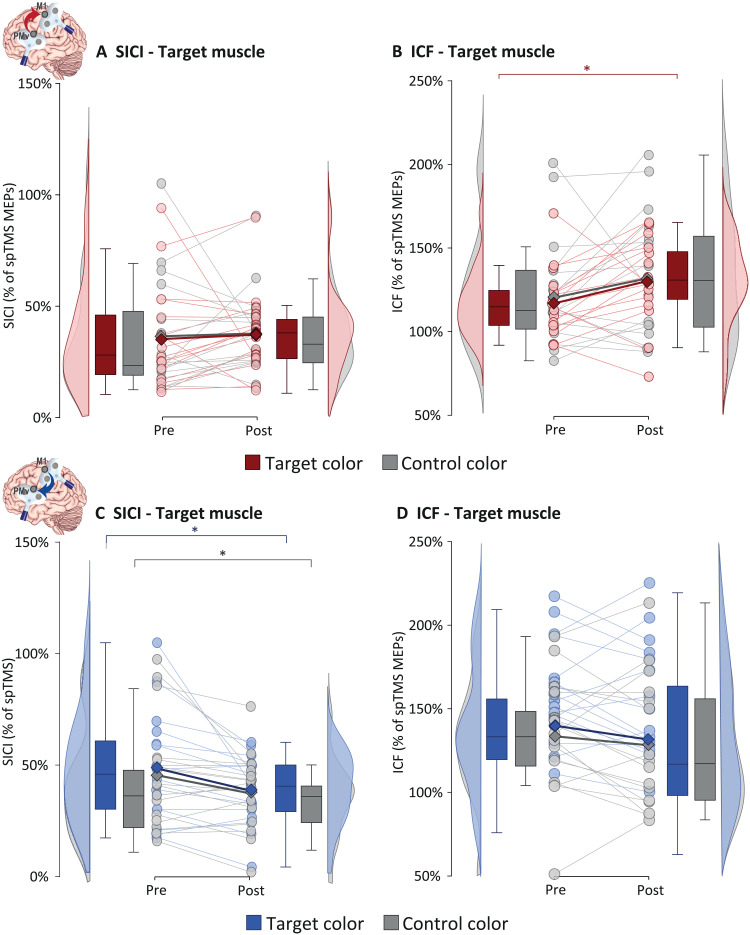
Results of experiment 1, testing the influence of state-dependent ccPAS on intracortical excitability indices. (**A**) No changes were observed for SICI following ccPAS_PMv-M1_. (**B**) ccPAS_PMv-M1_ increased ICF in response to the target visual cue. (**C**) ccPAS_M1-PMv_ increases the magnitude of SICI irrespective of the presented visual cue. (**D**) No changes were observed for the ICF index. Lighter circles and lines denote individual data points; darker squares and lines denote group means. Box plots represent the median, first and third quartiles, and the 95% confidence interval of individual data points. spTMS, single pulse TMS. Asterisks indicate significant post hoc comparison: *P < 0.05.

In experiment 1, ccPAS_PMv-M1_ was accompanied by a general shift toward increased ICF in the target muscle (Fig. 5B), significant for the presentation of the target color (Δ = 13%, *P* = 0.049, *r* = 0.49) but not the control one (*P* ≥ 0.21). No SICI modulations were observed (both *P* ≥ 0.13; Fig. 5A). Conversely, while the ccPAS_M1-PMv_ did not affect ICF (all *P* ≥ 0.25; Fig. 5D), we observed an enhanced SICI irrespective of the visual input presented (Fig. 5C), with similar changes for the target (Δ = 10%, *P* = 0.017, *r* = 0.59) and the control color (Δ = 8%, *P* = 0.034, *r* = 0.53). These findings indicate that ccPAS_PMv-M1_ facilitated and ccPAS_M1-PMv_ reduced CSE by acting on excitatory glutamatergic and GABAergic intracortical networks underlying the ICF and SICI metrics (*49*). Thus, the two ccPAS protocols appear to induce distinct changes in intracortical excitability: ccPAS_PMv-M1_ led to an increase in glutamate-mediated ICF, whereas ccPAS_M1-PMv_ resulted in an increase in GABAa-mediated intracortical inhibition. In experiment 2, we observed no significant training-induced changes in ICF or SICI (all *P* ≥ 0.08), suggesting that intracortical modulations were specific to state-dependent ccPAS.

### Behavioral relevance of state-dependent ccPAS to AVMM

**Fig. 6.**
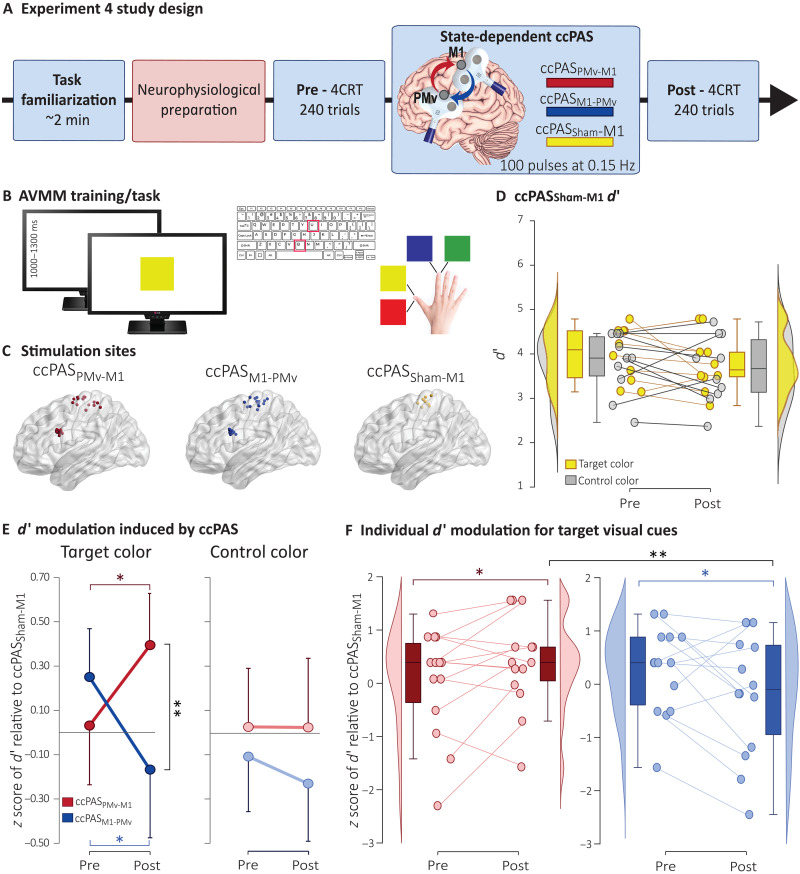
Methodology and results of experiment 4 testing the influence of state-dependent ccPAS on AVMM performance. (**A**) Design. (**B**) AVMM training—task. Participants were instructed to respond to two visual cues (one target and one control) with their index finger and to two different visual cues (one target and one control) with their thumb finger. The four cues appeared randomly, for a total of 90 trials for each color (see Materials and Methods for details). During the state-dependent ccPAS protocol, paired pulses were administered at the onset of target movements (i.e., during motor responses to target cues). No TMS pulses were delivered during control movements (i.e., during responses to control cues). (**C**) Individual targeted sites reconstructed on a standard template using MRIcron software (MRIcron/NPM/dcm2nii) after conversion to MNI space for illustrative purposes. (**D**) *d*′ performance in the ccPAS_Sham-M1_ group did not change over time when responding to either the target or the control color. (**E**) ccPAS_PMv-M1_ and ccPAS_M1-PMv_ induced opposite effects on *d*′ selectively in responding to target stimuli but not control ones. (**F**) Individual data points of *d*′ modulation in responding to target stimuli in the ccPAS_PMv-M1_ and ccPAS_M1-PMv_ sessions. Error bars represent one SD; box plots represent the median, first and third quartiles, and the 95% confidence interval of individual data points. *P < 0.05 and **P < 0.01.

Building on these neurophysiological findings, in experiment 4, we tested the behavioral impact of state-dependent ccPAS on AVMM in a new group of 24 healthy young adults. We used a modified AVMM task where participants responded to four visual cues (red, yellow, blue, or green squares) using two fingers (Fig. 6, A and B). Thus, for each participant, four counterbalanced AVMM associations were created, two for each finger. State-dependent ccPAS was applied during two target visuomotor associations, one for each finger, while two associations served as controls. Fourteen participants were tested in two separate sessions, one for the ccPAS_PMv-M1_ and the other for the ccPAS_M1-PMv_ protocol. Like experiment 3, a further control condition combining the AVMM task while receiving a ccPAS_Sham-M1_ was implemented in the remaining participants. This protocol ensured that any potential effects of ccPAS over the PMv-M1 circuit were not due to the repeated stimulation of M1 during task execution but rather to the manipulation of connectivity between PMv and M1.

To assess the effect of the combined intervention, all participants performed a two-choice response time (2CRT) task based on the same established AVMM rules. We used a more complex AVMM task than in experiments 1 to 3 to increase sensitivity to ccPAS, as simpler tasks often result in little behavioral changes due to ceiling effects (*29*, *50*). Because ccPAS effects on behavior can take longer to emerge compared to physiological effects (*29*, *30*, *50*), response times (RTs) and accuracy were collected as performance measures before (pre) and 30 min post-ccPAS intervention (post) (Fig. 6A). Accuracy was converted into measures of sensitivity (*d*′) and response bias (criterion) in accordance with signal detection theory (*51*). Separate *d*′ and criterion values were computed for the two target visual cues and, separately, for the two control visual cues, enabling statistical comparison of motor performance between trial types (see the “Data handling” section).

No significant effects of the ccPAS protocol emerged for RTs or criterion values (Supplementary Results, fig. S4, and tables S3 and S4). Conversely, significant ccPAS-induced modulations were observed in *d*′ indices of performance. The ccPAS_Sham-M1_ group displayed no performance modulations (all *P* ≥ 0.17; Fig. 6D); thus, to minimize unspecific TMS or practice effects, data from the ccPAS_PMv-M1_ and ccPAS_M1-PMv_ conditions were *z* score sham-corrected using data from the control ccPAS_Sham-M1_ group (*52*) and submitted to a session (ccPAS_PMv-M1_ and ccPAS_M1-PMv_) × time (pre and post) × color (target and control) ANOVA, revealing the significance of the three-way interaction (*F*_1,13_ = 4.89; *P* = 0.045; η_p_^2^ = 0.273; Fig. 6E). While ccPAS_PMv-M1_ induced an increase in performance, selectively for the target color (Δ = +0.36; *P* = 0.037; *d*_rm_ = 0.51), ccPAS_M1-PMv_ determined a selective decline in performance for the target color (Δ = −0.42; *P* = 0.025; *d*_rm_ = 0.48), and the *d*′ of the target color at post differed between the two ccPAS sessions (Δ = 0.56; *P* = 0.005; *d*_rm_ = 0.61). On the contrary, no modulation was observed in the control color following either ccPAS intervention (all *P* ≥ 0.45). In sum, these findings point to similar visuomotor-specific effects of state-dependent ccPAS on neurophysiology and behavior, inducing changes in performance of a simple behavioral AVMM task that are (i) bidirectional and (ii) more pronounced for the targeted visuomotor associations.

## DISCUSSION

In this study, we explored the plasticity and functional significance of the PMv-to-M1 pathway in AVMM. Experiments 1 and 4 used a state-dependent ccPAS approach designed to target neural populations encoding specific visuomotor associations within the PMv-M1 pathway. By asking participants to learn specific finger movements in response to target visual cues, we preconditioned the functional state of the subpopulation of visuomotor neurons encoding those visuomotor associations, making these neurons more receptive to TMS (*22*, *24*). The state-dependent involvement of PMv neurons in AVMM is supported by seminal evidence in monkeys (*7*–*9*). PMv projects to M1 via glutamatergic pathways, with most projections synapsing onto both glutamatergic and GABAergic interneurons in M1, which modulate the output of pyramidal cells, resulting in both excitatory and inhibitory effects on CSE (*53*–*56*). Accordingly, prior human studies show that during visually guided hand actions, the PMv exerts muscle-specific facilitation of M1 neurons (*37*, *38*, *57*). Moreover, during and before motor tasks, CSE increases for the moving muscles but can decrease in neighboring muscles (*58*). Building on this evidence and by manipulating the activity of specific neurons at the time of the PMv-M1 stimulation through a behavioral AVMM task, we aimed to achieve high functional specificity of ccPAS, thereby enhancing the potential for modifying synaptic strength within the PMv-M1 pathway in a functionally specific manner (*22*, *32*, *33*). Our results demonstrate that state-dependent ccPAS_PMv-M1_ and ccPAS_M1-PMv_ resulted in bidirectional influences in CSE and behavioral response to target visual cues, with distinct modulations of glutamatergic and GABAergic intracortical networks, providing insights into the physiological mechanisms and causal relevance of specific populations of neurons within the PMv-M1 pathway in AVMM (*29*, *30*, *35*, *36*, *44*, *45*, *50*, *59*, *60*). ccPAS builds upon the classical paired associative stimulation protocol (*61*) by delivering two TMS pulses to anatomically connected cortical sites with precise stimulus timing (*21*). The ccPAS protocol is believed to engage Hebbian STDP (*22*–*26*), where repeated and consistent activation of presynaptic neurons immediately before postsynaptic neurons activation promotes LTP. Conversely, when postsynaptic neurons are activated before presynaptic ones, LTD is typically induced (*26*–*28*, *40*, *41*). The ccPAS protocol simulates pre- and postsynaptic neuronal activation by delivering two TMS pulses to cortical areas in a precise temporal order with a specific ISI tailored to the anatomical and temporal dynamics of the targeted pathway (*21*). In line with these principles, previous studies have demonstrated ccPAS efficacy by applying it offline to manipulate PMv-M1 connectivity (*36*, *45*, *59*), cortical oscillatory activity (*59*, *60*), CSE (*35*, *44*), and motor functions (*29*, *30*, *44*, *50*). ccPAS_PMv-M1_ has consistently yielded LTP-like aftereffects (*29*, *30*, *35*, *36*, *42*, *44*, *45*, *50*), while ccPAS_M1-PMv_ has produced either inhibitory (*30*, *36*, *42*) or null results (*29*, *35*, *43*). Here, we leveraged the state-dependent properties of TMS to refine the ccPAS_PMv-M1_ and ccPAS_M1-PMv_ protocols by integrating a concurrent AVMM task. We designed this approach to enhance the functional specificity of the stimulation and evaluated the neurophysiological and behavioral outcome of manipulating functionally specific neurons within the PMv-M1 pathway. Moreover, experiments 2 and 3 tested the specificity of the ccPAS manipulation, further clarifying its physiological bases.

### State-dependent ccPAS_PMv-M1_ enhances visuomotor specificity and glutamatergic transmission

In experiment 1, state-dependent ccPAS_PMv-M1_ resulted in a finely tuned neuromodulation of CSE: Instead of the generic LTP-like effects commonly reported following offline ccPAS_PMv-M1_ (*29*, *30*, *35*, *36*, *45*, *50*, *59*, *62*), we observed a high visuomotor selectivity: We found increased CSE only in the target muscle that was active during the paired stimulation, and this response was maximal for the target color—although an increase of smaller magnitude was found also for the control color (see below). Conversely, in experiment 2, the AVMM training alone resulted in a generic and comparable increase of CSE for both moving muscles, with no influence of visual cues, likely reflecting plastic changes due to the motor component of the training (*63*–*65*). State-dependent ccPAS_PMv-M1_ acted on these neuroplastic effects by enhancing specific visuomotor neurons within the PMv-M1 pathway during the AVMM, resulting in a biased CSE modulation toward the color-movement combination marked by the PMv-M1 stimulation, thus increasing the specificity of the neuroplastic effects induced by AVMM training alone, allowing the influence of the targeted color to be detected in the CSE of the target muscle representation.

Moreover, in experiment 1, we observed an increase in ICF (but not SICI) induced by that state-dependent ccPAS_PMv-M1_. The ICF is a complex measure of intracortical excitation, thought to be influenced by glutamatergic facilitation through NMDA receptors and GABAergic inhibition through GABA_A_ receptors (*49*, *66*, *67*). The latter also contribute to the SICI index (*49*, *67*). Thus, the increase of ICF in the absence of SICI modulation suggests that state-dependent manipulation of PMv-M1 connectivity enhanced local glutamatergic activity. This is coherent with the role of this neurotransmitter on plasticity induction (*68*) and the notion that PMv neurons can exert inhibitory or excitatory effects on M1 interneuron networks depending on the ongoing functional state (*36*–*38*, *57*). The present changes in excitatory motor networks also appear specific to state-dependent activation of specific PMv-M1 circuits during ccPAS_PMv-M1_, as prior work using offline ccPAS_PMv-M1_ administered at rest reported modulation of SICI rather than ICF (*35*). Moreover, no consistent changes in ICF (or SICI) were found in experiment 2, further supporting the specificity of the state-dependent ccPAS_PMv-M1_ manipulation. These findings highlight the malleability and physiological impact of function-specific PMv-M1 neurons transforming visual information into motor output.

### State-dependent M1 activation contributes to motor selectivity of state-dependent ccPAS_PMv-M1_

Experiments 1 and 2 also indicate that state-dependent ccPAS_PMv-M1_ (experiment 1) suppressed plastic changes in the CSE of the control muscle that would have otherwise been induced by the AVMM training alone (experiment 2). Notably, experiment 3 clarified that this disruption of plasticity in nontargeted motor networks is likely due to the stimulation of M1 itself rather than the manipulation of PMv-M1 connectivity: The combination of AVMM training and M1 stimulation (ccPAS_Sham-M1_) in experiment 3 yielded a similar disruption of plasticity in the control muscle. This is consistent with previous evidence that TMS over M1 interleaved with a motor task can disrupt its motor consolidation (*69*, *70*). On the other hand, experiment 3 demonstrated increased CSE in the target muscle, aligning with prior findings that state-dependent M1 stimulation during a motor task can induce muscle-specific LTP-like enhancements in CSE (*46*). Thus, state-dependent activation of M1 alone can contribute to the motor selectivity observed in experiment 1, either in conjunction or independently of the activation of specific PMv-M1 connections.

In experiment 1, we also found that state-dependent ccPAS_PMv-M1_ increased CSE in the target muscle not only for target colors but also for control colors. This increase may reflect motor selective but visually nonspecific changes driven by TMS applied during target muscle movement (*46*). A similar effect was observed in experiment 3, where stimulation of M1 alone induced muscle-specific CSE facilitation for both target and control colors. This suggests that both state-dependent ccPAS_PMv-M1_ and ccPAS_Sham-M1_ likely produce muscle-specific CSE facilitation in response to any visual cue, attributable to the state-dependent stimulation of M1 neurons during movement execution. However, only state-dependent ccPAS_PMv-M1_ resulted in a biased CSE response dependent on the visual cue’s color, with the greatest CSE observed in response to the target cue, compared to the control cue. Thus, comparing the results from experiments 1 and 3 highlights that the distinctive effect of state-dependent ccPAS_PMv-M1_ lies in its ability to achieve visuomotor specificity by modulating PMv-to-M1 connectivity.

Together, findings from experiments 1 to 3 indicate that state-dependent ccPAS_PMv-M1_ specifically boosts the influence of targeted PMv-M1 neurons involved in visuomotor transformation, leading to visuomotor specificity of LTP-like CSE modulations and enhanced excitatory glutamatergic transmission in motor networks. In addition, the protocol induces a pattern of motor selectivity that mirrors—and is likely driven by—the simple state-dependent engagement of M1 neurons alone, involving LTP enhancement of plasticity in target motor networks and the concurrent dampening of plasticity in spatially overlapping nontarget motor networks.

### State-dependent ccPAS_PMv-M1_ reveals a causal role of function-specific PMv-M1 neurons in AVMM

Experiment 4 highlighted the behavioral relevance of function-specific PMv-M1 neurons in AVMM. We introduced more colors to increase stimulus and behavioral variability while maintaining the task intentionally simple, to align with the AVMM training used in experiments 1 to 3. As a result, the observed effects were small in magnitude but consistent in direction. Specifically, following state-dependent ccPAS_PMv-M1_ participants demonstrated improved sensitivity—indexed by higher *d*′ scores—in responding to target visual cues, with no changes in sensitivity observed for control visual cues. These findings were not due to changes in response bias or speed accuracy trade-off, as no corresponding effects were observed in the criterion or RTs. This indicates that the observed improvements in the ability to discriminate between the two target colors were genuine and not merely a result of altered decision criteria or participants slowing down their responses to prioritize accuracy over speed. All in all, these state-dependent ccPAS_PMv-M1_ findings highlight the plastic malleability and behavioral relevance to AVMM of function-specific PMv-M1 neurons encoding specific visuomotor associations.

### Physiological bases and behavioral impact of state-dependent ccPAS_M1-PMv_

In stark contrast, state-dependent ccPAS_M1-PMv_ yielded a markedly different set of physiological and behavioral results. In this case, during the AVMM training, we applied a reversed order of paired-pulse stimulation to disrupt the strength of PMv-M1 connectivity (*36*, *59*), in accordance with the Hebbian rule (*26*–*28*). We hypothesized that M1-PMv stimulation would counteract the hierarchical sequence of visuomotor transformation—from visual processing of target color to the motor activation of target muscle—and induce inhibitory effects in the physiological communication from PMv to M1 neurons during the AVMM training. Consistent with these hypotheses, in experiment 1, state-dependent ccPAS_M1-PMv_ resulted in a reduction of CSE for both the target and control colors. The inhibitory modulation was more pronounced for the target visual cues, indicating visuomotor selectivity.

CSE changes were observed in the target muscle only, indicating that both state-dependent ccPAS_PMv-M1_ and ccPAS_M1-PMv_ induced motor-selective aftereffects: They modulated motor networks that were engaged at the time of paired stimulation during the AVMM (target muscle) and disrupted the plasticity of spatially overlapping nontargeted motor networks (control muscle) that would have otherwise led to enhanced CSE (experiment 2). Thus, as for state-dependent ccPAS_PMv-M1_, the lack of CSE modulation in the control muscle following state-dependent ccPAS_M1-PMv_ was likely driven by the state-dependent activation of M1 rather than the manipulation of PMv-M1 connectivity, as suggested by experiment 3.

State-dependent ccPAS_M1-PMv_ modulation consisted of a strong reduction of CSE, accompanied by an increased SICI, indicating LTD-like effects driven by enhanced GABAergic transmission (*49*, *67*). While these findings generally align with previous studies that emphasize the role of GABA_A_ activity in learning and plasticity (*71*–*74*), they might be specific to the state-dependent application of ccPAS_M1-PMv_. As already discussed, AVMM training alone (experiment 2) or in combination with ccPAS_Sham-M1_ (experiment 3) resulted in excitatory rather than inhibitory aftereffects. Moreover, while a few studies have shown reduced CSE during the offline application of ccPAS_M1-PMv_ at rest (*30*), most of the literature reports no changes in CSE (*35*, *36*, *42*, *44*) or SICI (*35*), suggesting that active AVMM performance in experiment 1 was crucial for activating plastic inhibitory mechanisms.

Neurophysiological modulations following state-dependent ccPAS_M1-PMv_ were coherently complemented by changes in AVMM performance. Specifically, the inhibitory modulation, which was more prominent for target visual cues in experiment 1, was paralleled by a corresponding decrease in task sensitivity for target visual cues in experiment 4. The performance decline cannot be ascribed to changes in response strategies, as no variation in response criterion was observed nor to a speed accuracy trade-off, since no significant changes in RTs were detected across the different ccPAS protocols. Instead, these findings suggest that the activating inhibition between PMv and M1 directly impaired the neural mechanisms underlying the accurate association of visual cues with motor responses, independent of any changes in response speed or decision criteria. These findings, therefore, demonstrate that PMv-M1 projections are functionally malleable in both forward (PMv-to-M1) and backward (M1-to-PMv) directions and causally essential for visuomotor transformation underlying proficient AVMM performance.

### Limitations and future directions

Our study has a few limitations that should be acknowledged. First, although previous research indicates that intervention timing can influence associative plasticity aftereffects (*75*), we did not control for the time of day. While this factor deserves investigation in future studies, it is unlikely to substantially affect our main findings. In experiments 1 and 4, we ensured consistent timing of ccPAS sessions for each participant. This consistency supports our interpretation that the distinct patterns observed following ccPAS_PMv-M1_ versus ccPAS_M1-PMv_ reflect genuine excitatory and inhibitory mechanisms with specific behavioral consequences (*29*, *30*, *35*, *36*, *42*, *44*, *45*, *50*), rather than timing artifacts. Second, our investigation focused solely on the PMv-M1 network, which has been extensively studied using ccPAS (*21*, *29*, *30*, *35*, *36*, *42*, *43*). Future research should extend our approach to other sensorimotor networks involved in AVMM. Examining the physiological and behavioral contributions of function-specific neural populations within these networks would enhance our understanding of AVMM mechanisms. Moreover, future research could broaden the application of state-dependent ccPAS to investigate its potential for modulating connectivity across a wider range of perceptual, motor, and cognitive processes. For instance, state-dependent ccPAS over the V5-V1 network during visual motion perception has shown enhanced performance specific to the direction of motion presented during ccPAS (*33*). Our findings expand on this visual specificity by revealing visuomotor specificity in AVMM and PMv-M1 stimulation. This suggests that state-dependent ccPAS can target connectivity in a functionally specific manner, shaped by the stimulation protocol, the concurrent behavioral task, and the network being targeted. Last, while ccPAS is designed to align with Hebbian associative plasticity principles, direct empirical evidence at the cellular level is needed to confirm the Hebbian nature of the STDP-like effects induced by ccPAS.

In conclusion, our study provides relevant mechanistic insights into the physiological basis of ccPAS and offers compelling causal evidence that the PMv-M1 pathway is an essential component of the dorsolateral motor stream instrumental for arbitrary visuomotor transformations (*11*, *12*, *45*, *55*, *76*, *77*). By leveraging state-dependent features of TMS (*22*, *31*, *32*) and STDP mechanisms underlying the application of ccPAS (*21*–*25*), our findings highlight the plasticity and critical involvement of spatially overlapping but function-specific neuronal populations in enhancing visuomotor transformation within the PMv-M1 pathway. These insights have notable implications for optimizing ccPAS protocols, potentially leading to more targeted interventions in both experimental and clinical settings, particularly in the study and rehabilitation of visuomotor functions.

## MATERIALS AND METHODS

### Experimental design

#### Participants

Seventy-two right-handed young healthy volunteers with normal or corrected-to-normal vision participated in the four experiments (see the Supplementary Materials for demographic analyses). The participants were distributed as follows: 16 in experiment 1 (9 females; mean age ± SD: 23.4 years old ± 2.6), 16 in experiment 2 (10 females; 24.1 years ± 3.0), 16 in experiment 3 (8 females; 25.1 years ± 1.2), and 24 in experiment 4 (16 females; 24.8 years ± 1.5). Sample sizes were estimated using G*Power, with the expected effect size set to 0.3, derived from a recent meta-analysis (*21*). The significance level (α) was set at 0.05 and the statistical power at 0.90. For an ANOVA with eight repeated measures in a single group, the recommended sample size was 14. In experiments 1 to 3, this number was increased to 16 to ensure full counterbalanced random assignment of the target visuomotor association during ccPAS. All participants provided informed consent and were screened to exclude any individuals with psychiatric, neurological, or cardiac conditions, ongoing pharmacological treatments, or any other condition that might lead to adverse reactions to TMS (*78*). The experimental procedures were performed in accordance with the Declaration of Helsinki and later amendments (*79*) and approved by the Bioethics Committee of the University of Bologna. The study was not preregistered. No adverse reactions or discomfort related to TMS was reported by participants or noticed by the experimenters.

#### Experiments 1 to 3 study design

Experiments 1 to 3 investigated the physiological aftereffects and specificity of state-dependent ccPAS, targeting the PMv and primary motor cortex (M1). Experiment 1 tested the effect of state-dependent PMv-to-M1 ccPAS (ccPAS_PMv-M1_) and M1-to-PMv ccPAS (ccPAS_M1-PMv_) in two separate sessions (mean distance between experimental sessions: 13.10 ± 7.95 days; range: 6 to 33 days).

Although there are no established guidelines for the duration of the effects of a single ccPAS session, this spacing was determined on the basis of empirical evidence showing that the effects of single repetitive TMS sessions dissipate within 72 hours [e.g., (*80*, *81*)] and prior ccPAS studies with similar designs, which used comparable minimum intersession intervals [e.g., (*82*, *83*)]. For participants who completed two sessions (experiments 1 and 4), the time of day was consistent within individuals across sessions. The protocols were administered in a “state-dependent” manner, with participants undergoing ccPAS paired stimulation while performing an AVMM task. During the task, participants were asked to perform an abduction of either the right index finger (activating the FDI) or the little finger (activating the ADM) in response to corresponding visual cues (a blue or an orange square). For each participant, a target visuomotor association (e.g., FDI activation in response to the blue square) and a control visuomotor association (e.g., ADM activation in response to the orange square) were designed, with assignments counterbalanced across participants. Crucially, the delivery of ccPAS paired pulses was exclusively triggered during trials involving the target visuomotor association (e.g., FDI activation, blue square), 500 ms after the onset of the target visual stimulus. At every iteration of the target visuomotor association, a single pair of TMS pulses was delivered. The effect of state-dependent ccPAS was evaluated by recording MEPs from the target and control muscles. MEPs were elicited using single-pulse and paired-pulse TMS over M1, while participants were presented with the target and control visual cues. MEP amplitudes were analyzed to assess CSE, SICI, and ICF before and after ccPAS.

Each session began with the setup of electrode montage and the determination of the optimal M1 scalp position related to the assigned target muscle (e.g., the FDI muscle). This was followed by the assessment of the rMT and stimulation intensity to produce MEPs amplitudes of ~1 mV (SI_1mV_) from the target muscle. This intensity ensured stable MEPs recordings from both the target and control muscles for all participants.

The testing phase started with a baseline test block (pre), involving assessments of CSE, SICI, and ICF. This was followed by neuronavigation to identify the PMv site and the calibration of TMS parameters for the ccPAS protocols. Afterward, participants underwent state-dependent ccPAS, followed by a posttreatment test block.

Experiments 2 and 3 shared the same AVMM task and general structure as experiment 1 but involved a single session each with a different plasticity induction protocol. Experiment 2 investigated the effects of the AVMM task alone (without concurrent ccPAS) on CSE, SICI, and ICF. Experiment 3 examined the effects of a control state-dependent ccPAS protocol combining the AVMM task with repeated M1 stimulation (ccPAS_Sham-M1_) on CSE.

*Test block procedures*. Participants were seated comfortably in a chair and instructed to keep their right hand relaxed while viewing a computer screen (53 cm by 30 cm) positioned approximately 80 cm away. The screen displayed a randomized sequence of target and control visual cues (a blue or orange square measuring 200 × 200 pixels). Each trial began with a black screen lasting between 3000 and 5000 ms, followed by a gray screen for 1000 ms; next, the visual cue appeared for 500 ms, after which the screen returned to gray for 2000 ms (Fig. 1D); TMS was delivered during the presentation of the visual cue, either 250 or 320 ms after its onset, resulting in a variable interval between two TMS pulse ranging from 6430 to 8570 ms. For each index (CSE, SICI, and ICF), 20 trials per color plus 4 catch trials (44 total) were presented. Catch trials involved displaying random numbers that participants were instructed to read aloud, to ensure they were paying attention to the screen. The order in which the indices were collected was counterbalanced across participants. In experiment 1, this order was kept constant for the two ccPAS sessions.

CSE was assessed by measuring the amplitudes of MEPs elicited by a single TMS pulse (spTMS) over M1 at an intensity of SI_1mV_. SICI and ICF were assessed by collecting MEPs elicited by pairs of TMS pulses delivered through the same coil over the left M1: The first pulse, a conditioning pulse, was administered at a subthreshold intensity of 80% of the rMT and was followed by a second, test pulse at a suprathreshold intensity of SI_1mV_; the interval between pulses was set at 3 ms for SICI and to 12 ms for ICF, in accordance with established protocols (*35*, *47*, *48*).

*AVMM training and plasticity induction*. Following the pretesting block, two arbitrary visuomotor associations were communicated to the participants. Each visual cue (either the blue or orange square) required the execution of a specific finger movement (either an index finger abduction involving the FDI muscle or a little finger abduction involving the ADM muscle). One visuomotor association served as the “target visuomotor association” and involved the response of the target muscle (for which the optimal scalp position and stimulation parameters were determined) to the presentation of the “target visual cue” (e.g., FDI contraction in response to the blue square); the other association served as the “control visuomotor association” (e.g., ADM contraction in response to the orange square). All these pairings were counterbalanced across participants. In experiments 1 and 3, ccPAS paired stimulation was administered during the occurrence of the target visual cue/target muscle response. In experiment 2, no TMS was administered.

During the AVMM training, participants kept their right hand on a table in front of them and focused on a computer screen displaying alternating visual cues (Fig. 1B). Each trial started with a gray screen displayed for a randomly variable duration of 1000 to 1500 ms, followed by a visual cue lasting 1000 ms and requiring responding with the associated finger movement. Motor responses to both target and control cues were monitored by an experimenter and recorded via EMG (see the Supplementary Materials for analyses). After each visual cue, the screen turned to gray for 1250 ms and then black for 1500 ms. This sequence was repeated 180 times, with alternating presentation of 90 target visual cues and 90 control visual cues.

In experiments 1 and 3, the ccPAS paired stimulation occurred 500 ms after the onset of the target visual cue, during the active contraction of the target muscle. The state-dependent ccPAS protocols consisted of 90 pairs of TMS pulses over PMv and M1, administered at a frequency of ~0.1 Hz over 15 min (*30*, *35*, *36*, *44*). In one session of experiment 1, participants underwent ccPAS_PMv-M1_, with the PMv pulse in the ccPAS protocol preceding the M1 pulse by an 8-ms ISI, aimed at activating short-latency PM-M1 connections (*35*, *38*, *39*); in the other session, the pulse order was reversed (ccPAS_M1-PMv_), with the M1 pulse preceding the PMv pulse by 8 ms. To minimize carry over effects, the two sessions were separated by at least 6 days (mean interval between experimental sessions ± SD: 13.10 ± 7.95 days; range: 6 to 33 days) and were presented in a counterbalanced order across participants. In experiment 3, participants underwent a state-dependent ccPAS_Sham-M1_ protocol (ccPAS_Sham-M1_), involving repeated pairs of sham stimulation of PMv and active stimulation of M1, separated by an 8-ms ISI. Sham stimulation was performed by tilting the PMv coil by 90° so that no current was induced in the brain. This protocol was used to ensure that any potential effects of active state-dependent ccPAS were not merely due to the repeated stimulation of M1 during task execution but rather to the manipulation of connectivity between PMv and M1. Across these protocols, M1 stimulation intensity was set to SI_1mV_, and PMv stimulation intensity was adjusted to 90% of the individual rMT (*29*, *30*, *35*). Studies using these stimulation parameters for recording MEPs to paired-pulse stimulation demonstrated the activation of excitatory PMv-M1 pathways at rest (*35*, *39*), and similarly, offline ccPAS studies demonstrated the efficacy of these parameters for driving LTP-like effects during and following ccPAS_PMv-M1_ (*29*, *35*, *44*). Conversely, a few studies demonstrated LTD-like effects during the reversed ccPAS_M1-PMv_ protocol (*30*, *36*), although most of the studies reported no consistent aftereffects following this protocol (*29*, *35*, *44*). Pulses were remotely triggered by a MATLAB script (MathWorks, Natick, USA). All participants tolerated the stimulation well.

#### Experiment 4 study design

In experiment 4, we tested the behavioral impact of state-dependent ccPAS. After a brief training phase (~2′), participants performed an AVMM 2CRT task before (pre) and 30 min (post) after the end of the protocol (Fig. 4A). RTs and overall accuracy (percentage of correct responses) were collected as performance measures. Fourteen participants participated in two counterbalanced sessions, undergoing ccPAS_PMv-M1_ in one session and ccPAS_M1-PMv_ on a different day (mean distance between experimental sessions: 11.30 ± 5.80 days; range: 7 to 41 days). The remaining 10 participants underwent a ccPAS_Sham-M1_ protocol (ccPAS_Sham-M1_) similar to the one used in experiment 3, where PMv stimulation was sham and M1 stimulation was active, to ensure that any potential effects of state-dependent ccPAS were not merely due to the repeated stimulation of M1 during task execution but rather to the manipulation of connectivity between PMv and M1.

*Test block procedure*. Participants were seated in front of a computer screen as in experiments 1 to 3 and were asked to respond to the presentation of visual cues by pressing two buttons on a keyboard. Four different visual cues (red, yellow, blue, or green squares) could be presented, and participants were instructed to respond to two of them (e.g., red and yellow) by pressing the on key with their right index finger and to respond to the remaining two cues (e.g., blue and green) by pressing another key with their right thumb, as quickly and as accurately as possible. Thus, four randomized and counterbalanced AVMM associations were created, two for each finger. Each testing block consisted of 240 trials (60 per color). Before the start of the experiment, participants familiarized themselves with the task for ~5 min. Each trial started with the presentation of a white screen (random duration between 1 and 1.3 s), followed by the visual cue, requiring participants to respond with their index finger or thumb based on the AVMM associations communicated at the beginning of the experiment (Fig. 4B). No RT limit was imposed, but outlier RTs were excluded from the analysis (total % excluded trials: 1.58 ± 0.42%; see the “Data handling” section).

*AVMM and plasticity induction*. Participants were instructed to perform an AVMM task using the same AVMM associations used in the 2CRT task. Two of the visual cues, one for each finger (e.g., red and green squares), acted as target cues and were marked by ccPAS paired-pulse stimulation. The other two cues (e.g., yellow and blue squares) acted as control visual cues and were not associated with TMS. To expose participants to the same amount of target and control visual cues during the AVMM training and, at the same time, not substantially alter the architecture of the ccPAS protocol, we organized trials as follows: the task started with 50 trials presenting the control visual cues (e.g., yellow and blue squares) in a randomized order, followed by 100 randomly presented target cues (e.g., red and green squares), and then again 50 control visual cues. In this way, state-dependent ccPAS consisted of a continuous block lasting ~11 min, ccPAS paired-pulse stimulation delivered at ~0.15 Hz, with the same critical 8-ms ISIs as in experiment 1. The response to the two target cues triggered the delivery of pairs of TMS pulses over PMv and M1. Each trial started with a black screen displayed for 2000 ms, followed by 2000 ms of gray screen. Then, the visual cue appeared, requiring the production of the associated key press, which triggered the paired TMS pulses when target cues were presented. The colored visual cue disappeared from the screen 250 ms after the production of the correct response, and the screen turned to gray for 2000 ms. Assuming an RT of ~400 ms, based on the behavioral performance of participants (fig. S3A), each trial lasted around 6650 ms. This sequence was repeated 200 times, resulting in a 22′ intervention with a ~0.15-Hz ccPAS.

#### TMS and EMG recording

In all experiments, TMS was performed using two 50-mm figure-of-eight coils connected to a Magstim Bistim^2^ stimulator (The Magstim Company, Carmarthenshire, Wales, UK). We used iron branding coils with the handle perpendicular to the plane of the wings to minimize the handles’ interference during ccPAS. The two Magstim 200^2^ modules of the Magstim Bistim^2^ were connected during the test blocks in experiments 1 to 3 and left unconnected for performing state-dependent ccPAS in experiments 1, 3, and 4. Because the output intensity can differ when using the two modules separately, the rMT and SI_1mV_ were assessed for both the connected and disconnected configurations. The relevant parameters were then used for the test blocks and state-dependent ccPAS. Reliability analyses indicated highly consistent rMT values across sessions for participants who took part in experiments 1 and 4, which comprised two sessions (fig. S1).

Ag/AgCl surface electrodes were placed using a belly-tendon montage, with ground electrodes placed on the right wrist. EMG signals were recorded using a Biopac MP-35 (Biopac, USA) electromyograph, band-pass–filtered between 30 and 500 Hz, sampled at 10 kHz, digitized, and stored for offline analysis. In all experiments, EMG activity was recorded from the muscles involved in the corresponding AVMM task: in experiments 1 to 3, these were the right FDI and ADM muscles; in experiment 4, the FDI and APB muscles.

The M1 coil was positioned over the optimal scalp position for eliciting maximal MEPs. In experiments 1 to 3, this position was determined on the basis of the assigned target muscle, with half of the participants being stimulated over the FDI representation and the other half over the ADM representation. In experiment 4, no distinction was made between target and control muscles since both fingers were used to respond to target and control cues, and the optimal position was selected to evoke maximal MEPs from both the FDI and APB muscles. The M1 coil was held tangentially to the scalp at an angle of 45° from the midline, to induce a posterior-to-anterior current in the brain (*84*). The rMT was defined as the minimum intensity of the stimulator output that induced MEPs with an amplitude of ≥50 μV in 5 of 10 consecutive trials (*85*). The SI_1mV_ was set to produce a MEP of ~0.75 to 1.25 mV. Across experiments 1 to 4, the rMT and SI_1mV_ were determined on the basis of the most excitable muscle, which corresponded to the target muscle in experiments 1 to 3. During state-dependent ccPAS in experiment 1, the PMv coil was placed tangentially to the scalp, inducing a direction of the current pointing toward the M1 site (*29*, *30*, *35*, *86*).

#### Neuronavigation

One coil was placed over the left M1, functionally localized at the start of the experiments as the optimal scalp position. The other coil placed over the left PMv, which was identified using the SofTaxic Navigator System (Electro Medical System, Bologna, IT). Skull landmarks (2 preauricular points, nasion and inion) and ~80 points were digitized using a Polaris Vicra digitizer (Northern Digital). We obtained an estimated magnetic resonance imaging (MRI) through a three-dimensional warping procedure fitting a high-resolution MRI template from each participant’s scalp and craniometric points. To target the left PMv, we used the following Talairach coordinates: *x* = −52; *y* = 10; *z* = 24, consistent with those used in previous studies (*34*, *37*, *87*). The Talairach coordinates corresponding to the projections of the left PMv and M1 scalp sites onto the brain surface were estimated by the SofTaxic Navigator from the MRI-constructed stereotaxic template, and the resulting coordinates are consistent with the regions defined as human PMv and M1 (Table.S2, Figs. 1C, 3, B and E, and 4E) (*88*).

### Statistical analysis

#### Data handling

Neurophysiological data were processed offline. MEP peak-to-peak amplitudes in millivolts were measured within a 60-ms window, starting 15 ms after the test TMS pulse, using a custom-made MATLAB script available at https://github.com/SoniaTurrini/MEPautomatedanalysis. Because background EMG affects motor excitability (*89*), MEPs preceded by background EMG activity deviating from the individual mean of the block by more than 2 SDs were discarded from the analysis; moreover, MEPs deviating from the mean amplitude of their test block by more than 3 SDs were also discarded (total % of excluded MEPs = 6.4 ± 2.3%) (*35*). For each subject, the muscle corresponding to the M1 optimal scalp position was labeled “target,” while the other was labeled as “control”; similarly, the color associated with the contraction of the target muscle was labeled as target, and the other as control, following the AVMM associations. For each condition, CSE was computed as the mean amplitude of MEPs induced by spTMS. SICI and ICF indices were calculated for each condition by dividing the mean MEP amplitudes elicited by paired-pulse TMS (conditioned and test pulse) by those elicited by spTMS (test pulse alone). To ensure that participants were correctly performing the AVMM task in experiments 1 to 3, we analyzed the EMG activity to assess the muscular contraction onset time and magnitude. Details are reported in the Supplementary Materials.

In experiment 4, we assessed AVMM task performance by measuring mean task accuracy (percentage of the correct response) and RTs in different conditions. RTs above or below 3 SDs from the mean of each condition were excluded from the analysis, to ensure that outlier responses did not affect the results. Accuracy was converted into measures of sensitivity (*d*′) and response bias (criterion) in accordance with signal detection theory (*51*). Separately for the two target colors and the two control colors, we considered a correct index response a “hit,” a correct thumb response a “correct rejection,” an incorrect index response a “false alarm,” and an incorrect thumb response a “miss.” For example, a participant could be instructed to respond with their index finger to red and yellow visual cues and with their thumb to blue and green visual cues. The target visual cues, whose presentation elicits paired TMS pulses, are red for the index finger and blue for the thumb; thus, the control visual cues are yellow for the index finger and green for the thumb. Values of the *d*′ and criterion were computed for target and control visual cues separately to be able to statistically compare the motor performance between trial types.

CSE data from experiment 1 were analyzed by means of an ANOVA with the following within-subjects factors: session (ccPAS_PMv-M1_ and ccPAS_M1-PMv_), time (two levels: pre and post), muscle (two levels: target and control), and color (two levels: target and control). This revealed the higher-order quadruple interaction (*F*_1,15_ = 8.45; *P* = 0.011; η_p_^2^ = 0.361) which was analyzed by running two separate repeated measures ANOVAs, one for the target muscle and the other for the control muscle. CSE data from experiments 2 and 3 were analyzed by means of an ANOVA with the following within-subjects factors: time (two levels: pre and post), muscle (two levels: target and control), and color (two levels: target and control).

The analyses conducted on SICI and ICF (experiments 1 and 2) concerned only MEPs recorded from the target muscle since the conditioning stimulus intensity was adjusted on the rMT assessed over the target muscle, making them the only reliable measure. We used nonparametric Wilcoxon’s tests, due to nonnormal distribution as assessed through the Lilliefors test.

Further analyses were conducted on the EMG traces recorded during the ccPAS protocol, to ensure similar contraction onset times, and amplitudes were observed across sessions and experiments. Details are reported in the Supplementary Materials.

In experiment 4, we first analyzed performance in the ccPAS_Sham-M1_ group. RTs were analyzed using an ANOVA with the following within-subjects factors: finger (index and thumb), color (target and control), and time (pre and post). *d*′ and criterion values were analyzed through two separate ccPAS (ccPAS_PMv-M1_ and ccPAS_M1-PMv_) by color (target and control) and by time (pre and post) ANOVAs. Then, data from the ccPAS_PMv-M1_ and ccPAS_M1-PMv_ conditions were sham-corrected and transformed into *z* scores to exclude unspecific TMS or practice effects (*52*) and submitted to three ANOVAs, one for each performance metric (RTs, *d*′, and criterion). The ANOVA on RTs included the within-subjects factors ccPAS (ccPAS_PMv-M1_ and ccPAS_M1-PMv_), color (target and control), finger (index and thumb), and time (pre and post). The ANOVAs on *d*′ and criterion values included the within-subjects factors ccPAS (ccPAS_PMv-M1_ and ccPAS_M1-PMv_), color (target and control), and time (pre and post).

For all ANOVAs, Duncan’s post hoc analyses were performed to correct for multiple comparisons. η_p_^2^ was computed as a measure of effect size for significant main effects and interactions; by convention, η_p_^2^ effect sizes of ~0.01, ~0.06, and ~0.14 are considered small, medium, and large, respectively. For significant within-subjects and between-subjects post hoc comparisons, we computed the appropriate Cohen’s *d* effect size (*d*_rm_ and *d*_s_, respectively); *d* effect sizes of ~0.2, ~0.5, ~0.8 are considered small, medium, and large (*90*). Effect sizes for nonparametric comparisons were estimated by approximating *z* scores to *r* (*91*). By convention, *r* effect sizes of ~0.1, ~0.3, and ~0.5 are considered small, medium, and large, respectively. All the analyses were conducted using STATISTICA version 12.

## Supplementary Materials

### This PDF file includes:

Supplementary Text Figs. S1 to S4 Tables S1 to S4 References
