# Supplementary material for: State-dependent associative plasticity highlights function-specific premotor-motor pathways crucial for arbitrary visuomotor mapping
Source: Sci Adv. 2025 May 14;11(20):eadu4098. doi: 10.1126/sciadv.adu4098 (PMC12077503; doi:10.1126/sciadv.adu4098)
Supplement: Supplementary file 1 — Supplementary Text Figs. S1 to S4 Tables S1 to S4 References [file sciadv.adu4098_sm.pdf]

Science Advances
AAAS

Supplementary Materials for

State-dependent associative plasticity highlights function-specific premotor-motor pathways crucial for arbitrary visuomotor mapping

Sonia Turrini *et al.*

Corresponding author: Sonia Turrini, sonia.turrini3@unibo.it; Alessio Avenanti, alessio.avenanti@unibo.it

Sci. Adv. **11**, eadu4098 (2025)
DOI: 10.1126/sciadv.adu4098

This PDF file includes:

Supplementary Text
Figs. S1 to S4
Tables S1 to S4
References

Supplementary Text

Demographic analyses

Age, sex and corticospinal excitability (CSE) as indexed by the resting motor threshold (rMT) value did not differ between individuals randomly allocated to the four Experiments (Table S1).

Coordinates for the targeted stimulation sites across groups can be found in Table S2, and are depicted in Fig. 1, 3 and 4 of the main text. To ensure that no difference occurred in stimulated sites between sessions in participants from Experiments 1 and 4, we ran multiple paired t tests comparing each coordinate in the two sessions and found no evidence of differences between the two sessions (all $p \geq .217$). Concerning coordinates of the M1 specifically, whose location was determined functionally at each session, we ran a reliability analysis to ensure individual values of all stimulation coordinates remained reasonably stable across sessions and observed significant intraclass correlation (ICC) measures for all 3 coordinates (x: two-way random ICC single measures = .618; CI = 0.337-0.798, $p < .001$; y: two-way random ICC single measures = .648; CI = 0.381-0.816, $p < .001$; z: two-way random ICC single measures = .455; CI = 0.119-0.697, $p = .005$). In Experiments 1 and 4, where participants were tested twice on two separate days, their rMT was highly reliable between sessions (two-way random ICC single measures = .848; CI = 0.726-0.925, $p < .001$, Fig. S1)

Experiments 1, 2 and 3: ccPAS EMG traces analysis

To ensure that participants were correctly performing the visuomotor mapping task during the ccPAS, as instructed, we analyzed the EMG trace recorded during the protocol to assess the muscular contraction onset time and magnitude. Data were processed offline. Muscle contraction onset times (OT) were extracted to ensure participants initiated the movement before each paired stimulation delivery; muscle contraction amplitude was extracted as an index of the fact that participants were correctly performing the visuomotor associations initially communicated (i.e., they were abducting the target finger/contracting the target muscle when viewing the target color, and abducting the control finger/contracting the control muscle when viewing the control color). All EMG analyses were conducted using custom-made MATLAB scripts.

Muscle contraction OT: For each of the 180 stimulus presentations (90 target + 90 control stimuli), the OT was calculated by moving a 20 ms window across the EMG data, starting from the stimulus presentation and sliding it forward in 1 ms increments. The standard deviation of the EMG signal within each window was calculated and compared to the standard deviation of the signal in the 100 ms before stimulus onset (the baseline period). Once the standard deviation of the data in the 20 ms window was over 2.75 times that of the baseline period for three successive 20 ms windows, the end of the first window was taken as the end of the OT period [92]. Additional visual inspection was performed to make sure that this time point accurately reflected the onset of the EMG response for every trial performed by every participant. Because for each trial only one of the two fingers was abducted and only one muscle contracted (i.e., the target muscle when presented with the target color, and the control muscle when presented with

the control color), for target color presentation only the onset time of the target muscle movement was extracted, and for control color presentation only the onset time of the control muscle movement was extracted.

Muscle contraction amplitude: For each of the 180 stimulus presentations (90 target + 90 control stimuli) the amplitude of muscle contraction was calculated as the mean of the rectified EMG trace of both the target and control muscles in two time windows of 400 ms each: a pre-stimulus window between -401 to -1 ms before the visual stimulus presentation, and a post-stimulus window between +1 to +401 ms from the visual stimulus presentation. Note that the post-stimulus window ends 99 ms before the TMS stimulation delivery, to make sure TMS-evoked activity does not contaminate our measure (Fig.S2). For each trial and visual stimulus presentation (i.e., both target and control color presentations), contraction was always recorded for both muscles (target and control). This allowed us to make sure that i) across trials, similar baseline muscle relaxation was obtained and ii) in each trial, only the finger associated with the presented visual stimulus was moved (and the corresponding muscle contracted), whereas the other one stayed relaxed.

Statistical analysis

To ensure the effects observed in Experiment 1 were not driven by discrepancies in muscle activations during ccPAS, we conducted one ANOVA on OT data with within factors Session (2 levels: $ccPAS_{PMv\text{-}M1}$, $ccPAS_{M1\text{-}PMv}$) and Muscle (2 levels: Target, Control) and one ANOVA on muscle contraction (rectified EMG mean) data with within factors Session (2 levels: $ccPAS_{PMv\text{-}M1}$, $ccPAS_{M1\text{-}PMv}$), Muscle (2 levels: Target, Control), Trial (2 levels: movement trial, non-movement trial) and Time (2 levels: Pre-stimulus, Post-stimulus). Tukey's post-hoc analyses were performed to correct for multiple comparisons. Partial η^2 (η_p^2) was computed as a measure of effect size for significant main effects and interactions. For significant post-hoc comparisons Cohen's *d* were computed. By convention, η_p^2 effect sizes of ~ .01, ~.06, and ~.14 are considered small, medium and large, respectively. All the analyses were conducted using STATISTICA version 10 and/or IBM SPSS Statistics version 25.

Similar analyses were performed for Experiments 2 and 3. Due to corruption of the large EMG files, we were unable to analyze 9 out of 64 traces (16 participants x 2 sessions in Experiment 1; 16 participants in Experiment 2; 16 participants in Experiment 3).

Results

OT: The analysis on movement onset times revealed no main effects nor interaction in Experiment 1 (all $F \leq 0.37$; all $p \geq .55$; Fig. S3, panel A), Experiment 2 ($F_{1,14} = 0.75$; $p = .402$; Fig. S3 panel C) and Experiment 3 ($F_{1,14} = 2.83$; $p = .115$; Fig. S3, panel E). This implies that, in both experiments and across experimental sessions, the target and control muscle were similarly reactive and engaged at similar latencies upon the presentation of visual cues.

Muscle contraction amplitude: The analysis on muscle contraction conducted on data collected in Experiment 1 revealed the main effects of Trial ($F_{1,11} = 26.85$; $p < .001$; $\eta_p^2 = 0.709$), and Time ($F_{1,11} = 47.44$; $p < .001$; $\eta_p^2 = 0.812$), further qualified by the interaction Trial x Time ($F_{1,11} = 53.31$; $p < .001$; $\eta_p^2 = 0.829$; Fig. S3, panel B). Post hoc tests were conducted to explore this interaction and revealed that muscle contraction differed between before and after the visual stimulus presentation only in movement trials ($p < .001$), but not in non-movement trials ($p = .351$). This means that the FDI muscle contracted after the presentation of the visual stimulus only in trials where the index finger was

supposed to move and, vice versa, the ADM muscle contracted after the presentation of the visual stimulus only in trials where the little finger was supposed to move.

Similar results were observed for Experiments 2 (Trial x Time interaction: $F_{1,14} = 26.64$; $p < .001$; $\eta_p^2 = 0.656$; Fig. S3, panel D) and Experiment 3 (Trial x Time interaction: $F_{1,14} = 32.57$; $p < .001$; $\eta_p^2 = 0.699$; Fig. S3, panel F). Post hoc tests were conducted to explore this interaction and revealed that muscle contraction differed between before and after the visual stimulus presentation only in movement trials (Experiment 2: $p < .001$, Experiment 3: $p < .001$), but not in non-movement trials (Experiment 2: $p = .377$; Experiment 3: $p = .572$).

In Experiment 3, we also observed a Muscle x Trial interaction ($F_{1,14} = 7.38$; $p = .017$; $\eta_p^2 = 0.345$), driven by a larger muscular contraction recorded in the target muscle as opposed to the control muscle in movement trials ($p = .013$).

Experiment 4: supplementary results

The analysis on RTs revealed the main effect of the factor Finger in the $ccPAS_{Sham\text{-}M1}$ group ($F_{1,9} = 6.051$; $p = .036$; $\eta_p^2 = 0.402$), which reflected higher response speed with the index compared to the thumb finger (Fig. S4). Sham-corrected RTs of the $ccPAS_{PMv\text{-}M1}$ or $ccPAS_{M1\text{-}PMv}$ groups revealed no effect of the stimulation protocol (all $p \geq .13$). The analysis of criterion values revealed no modulation in the $ccPAS_{Sham\text{-}M1}$ group (all $p \geq .07$). Sham-corrected criterion data of the $ccPAS_{PMv\text{-}M1}$ or $ccPAS_{M1\text{-}PMv}$ groups also showed no significant modulation (all $p \geq .10$).

Figures and Tables

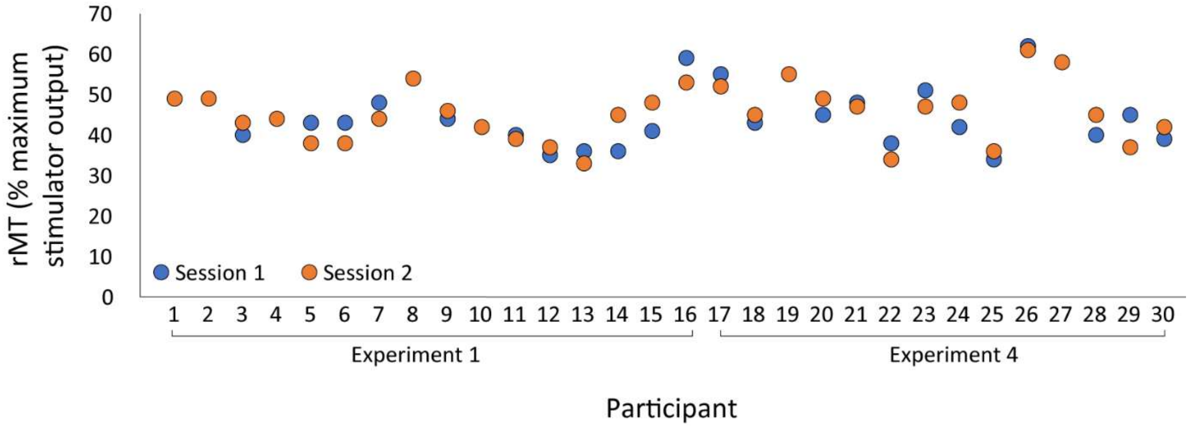

Figure S1. Intraclass correlation of rMT values for Experiments 1 and 4, where participants were tested twice.

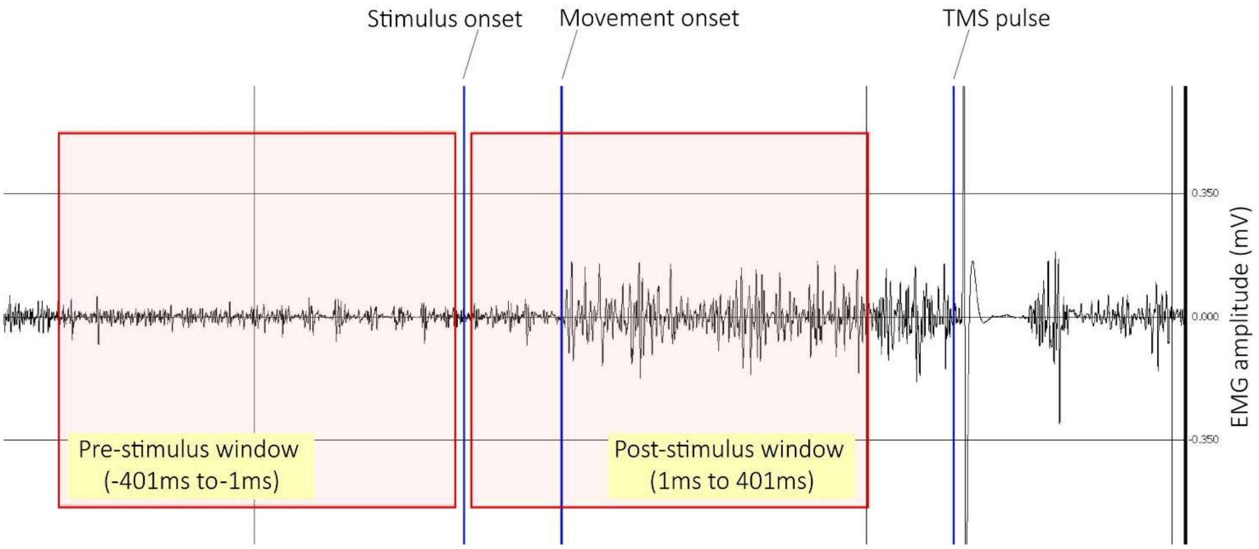

Figure S2. Example of the EMG trace of one trial, in one participant.

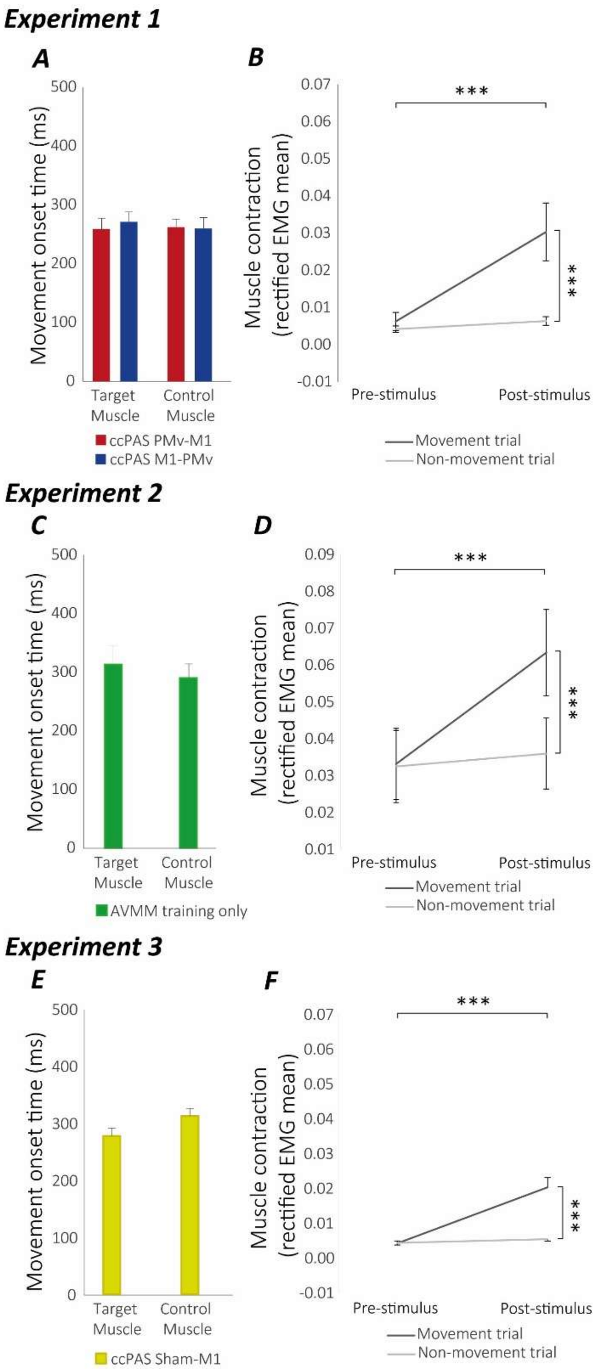

Figure S3. Movement onset and muscular contraction during the AVMM training in Experiments 1, 2, and 3. **A)** Movement onset time did not differ between muscles or sessions in Experiment 1. **B)** In both sessions of Experiment 1, in both muscles the contraction increased after the stimulus presentation only in trials when the corresponding finger was supposed to move. **C)** Movement onset time did not differ between muscles in Experiment 2. **D)** In Experiment 2, in both muscles the contraction increased after the stimulus presentation only in trials when the corresponding finger was supposed to move. **E)** Movement onset time did not differ between muscles in Experiment 3. **F)** In Experiment 3, in both muscles the contraction increased after the stimulus presentation only in trials when the corresponding finger was supposed to move. Error bars represent one SEM. ***$p<.001$.

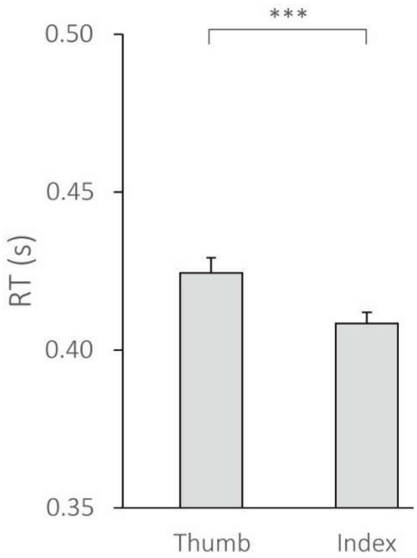

Figure S4. Main effect of finger on movement onset times. Participants were faster when responding with their index rather than the thumb finger. Error bars represent one SEM. ***p<.001

				rMT	Age	Gender
Experiment 1	ccPAS PMv-M1		*Avg*	43.9	23.4	M/F = 7/9
	ccPAS M1-PMv		*SD*	6.6	2.6	
Experiment 2			*Avg*	51.5	24.1	M/F = 6/10
			SD	11.4	3.0	
Experiment 3			*Avg*	47.6	25.5	M/F = 8/8
			SD	7.1	5.0	
Experiment 4	ccPAS PMv-M1		*Avg*	46.8	25.4	M/F = 5/9
	ccPAS M1-PMv		*SD*	8.3	1.3	
	ccPAS Sham-M1		*Avg*	44.6	23.4	M/F = 3/7
			SD	7.1	1.2	
Statistical comparison				*F=2.05; p>.09*	*F=1.35; p>.26*	*X2=1.32; p>.85*

Table S1. *Age, rMT and sex balance of participants who took part in the four Experiments.*

			M1			PMv		
			x	y	z	x	y	z
Experiment 1	ccPAS PMv-M1	*Avg*	-31.7	-20.7	59.3	-53.4	8.9	24.4
		SD	6.7	6.9	5.0	2.3	1.3	1.0
	ccPAS M1-PMv	*Avg*	-31.7	-19.1	60.2	-53.7	9.2	24.0
		SD	4.3	4.4	2.2	1.8	2.3	1.3
Experiment 2		*Avg*	-28.7	-17.5	60.6	/	/	/
		SD	6.8	4.8	5.5	/	/	/
Experiment 3		*Avg*	-33.8	-26.4	57.6	/	/	/
		SD	8.8	10.1	4.4	/	/	/
Experiment 4	ccPAS PMv-M1	*Avg*	-30.5	-18.7	60.5	-55.0	11.7	24.1
		SD	8.4	11.0	4.6	3.7	2.2	1.9
	ccPAS M1-PMv	*Avg*	-31.9	-17.7	61.8	-54.3	10.7	23.1
		SD	11.59	9.20	5.95	4.56	2.30	1.86
	ccPAS Sham-M1	*Avg*	-26.3	-18.0	59.3	/	/	/
		SD	4.9	4.8	4.4	/	/	/

Table S2. *Coordinates of the targeted cortical sites across the four studies.*

		Criterion (c)			
		Target Color		Control Color	
		Pre	*Post*	*Pre*	*Post*
ccPAS PMv-M1	*Average*	-0.17	-0.03	-0.02	-0.05
	SD	0.25	0.22	0.16	0.20
ccPAS M1-PMv	*Average*	-0.04	-0.06	0.03	0.06
	SD	0.21	0.18	0.21	0.33
ccPAS Sham-M1	*Average*	-0.06	0.00	0.05	-0.03
	SD	0.28	0.28	0.22	0.14

Table S3– *Average and standard deviation of criterion values across timepoints in all groups.*

		RTs							
		Target Color				Control Color			
		Thumb		*Index*		*Thumb*		*Index*	
		Pre	***Post***	***Pre***	***Post***	***Pre***	***Post***	***Pre***	***Post***
ccPAS PMv-M1	*Average*	479	454	467	437	495	463	489	436
	SD	62	50	56	41	90	54	101	38
ccPAS M1-PMv	*Average*	502	471	483	454	510	479	487	455
	SD	104	89	90	81	115	105	84	79
ccPAS Sham-M1	*Average*	451	429	428	407	431	418	425	408
	SD	36	32	37	33	27	35	34	24

Table S4– *Average and standard deviation of RTs, expressed in ms, across timepoints in all groups.*

REFERENCES AND NOTES

1. S. P. Wise, E. A. Murray, Arbitrary associations between antecedents and actions. *Trends Neurosci.* **23**, 271–276 (2000).

2. U. Halsband, R. Passingham, The role of premotor and parietal cortex in the direction of action. *Brain Res.* **240**, 368–372 (1982).

3. M. Petrides, Motor conditional associative-learning after selective prefrontal lesions in the monkey. *Behav. Brain Res.* **5**, 407–413 (1982).

4. A. Brovelli, D. Chicharro, J.-M. Badier, H. Wang, V. Jirsa, Characterization of cortical networks and corticocortical functional connectivity mediating arbitrary visuomotor mapping. *J. Neurosci.* **35**, 12643–12658 (2015).

5. E. A. Murray, S. P. Wise, Role of the hippocampus plus subjacent cortex but not amygdala in visuomotor conditional learning in rhesus monkeys. *Behav. Neurosci.* **110**, 1261–1270 (1996).

6. I. Toni, N. Ramnani, O. Josephs, J. Ashburner, R. E. Passingham, Learning arbitrary visuomotor associations: Temporal dynamic of brain activity. *Neuroimage* **14**, 1048–1057 (2001).

7. A. R. Mitz, M. Godschalk, S. P. Wise, Learning-dependent neuronal activity in the premotor cortex: Activity during the acquisition of conditional motor associations. *J. Neurosci.* **11**, 1855–1872 (1991).

8. N. Zach, D. Inbar, Y. Grinvald, H. Bergman, E. Vaadia, Emergence of novel representations in primary motor cortex and premotor neurons during associative learning. *J. Neurosci.* **28**, 9545–9556 (2008).

9. R. Romo, A. Hernández, A. Zainos, Neuronal correlates of a perceptual decision in ventral premotor cortex. *Neuron* **41**, 165–173 (2004).

10. K. Kurata, D. S. Hoffman, Differential effects of muscimol microinjection into dorsal and ventral aspects of the premotor cortex of monkeys. *J. Neurophysiol.* **71**, 1151–1164 (1994).

11. L. Fogassi, V. Gallese, G. Buccino, L. Craighero, L. Fadiga, G. Rizzolatti, Cortical mechanism for the visual guidance of hand grasping movements in the monkey: A reversible inactivation study. *Brain* **124**, 571–586 (2001).

12. G. Rizzolatti, L. Cattaneo, M. Fabbri-Destro, S. Rozzi, Cortical mechanisms underlying the organization of goal-directed actions and mirror neuron-based action understanding. *Physiol. Rev.* **94**, 655–706 (2014).

13. M. Davare, M. Andres, G. Cosnard, J. L. Thonnard, E. Olivier, Dissociating the role of ventral and dorsal premotor cortex in precision grasping. *J. Neurosci.* **26**, 2260–2268 (2006).

14. L. F. Schettino, S. V. Adamovich, H. Bagce, M. Yarossi, E. Tunik, Disruption of activity in the ventral premotor but not the anterior intraparietal area interferes with on-line correction to a haptic perturbation during grasping. *J. Neurosci.* **35**, 2112–2117 (2015).

15. A. Zangrandi, A. Mioli, M. D'Alonzo, D. Formica, G. Pellegrino, G. Di Pino, Conditioning transcranial magnetic stimulation of ventral premotor cortex shortens simple reaction time. *Cortex* **121**, 322–331 (2019).

16. N. Dancause, S. Barbay, S. B. Frost, E. J. Plautz, M. Popescu, P. M. Dixon, A. M. Stowe, K. M. Friel, R. J. Nudo, Topographically divergent and convergent connectivity between premotor and primary motor cortex. *Cereb. Cortex* **16**, 1057–1068 (2006).

17. S. Ghosh, R. Gattera, A comparison of the ipsilateral cortical projections to the dorsal and ventral subdivisions of the macaque premotor cortex. *Somatosens. Mot. Res.* **12**, 359–378 (1995).

18. E. Dayan, L. G. Cohen, Neuroplasticity subserving motor skill learning. *Neuron* **72**, 443–454 (2011).

19. F. Hamzei, C. H. Läppchen, V. Glauche, I. Mader, M. Rijntjes, C. Weiller, Functional plasticity induced by mirror training: The mirror as the element connecting both hands to one hemisphere. *Neurorehabil. Neural Repair* **26**, 484–496 (2012).

20. B. A. Philip, S. H. Frey, Increased functional connectivity between cortical hand areas and praxis network associated with training-related improvements in non-dominant hand precision drawing. *Neuropsychologia* **87**, 157–168 (2016).

21. P. D. Di Luzio, L. Brady, S. Turrini, V. Romei, A. Avenanti, A. Sel, Investigating the effects of cortico-cortical paired associative stimulation in the human brain: A systematic review and meta-analysis. *Neurosci. Biobehav. Rev.* **167**, 105933 (2024).

22. V. Romei, G. Thut, J. Silvanto, Information-based approaches of noninvasive transcranial brain stimulation. *Trends Neurosci.* **39**, 782–795 (2016).

23. G. Koch, Cortico-cortical connectivity: The road from basic neurophysiological interactions to therapeutic applications. *Exp. Brain Res.* **238**, 1677–1684 (2020).

24. D. Pitcher, B. Parkin, V. Walsh, Transcranial magnetic stimulation and the understanding of behavior. *Annu. Rev. Psychol.* **72**, 97–121 (2021).

25. L. Tarasi, S. Turrini, A. Sel, A. Avenanti, V. Romei, Cortico-cortical paired-associative stimulation to investigate the plasticity of cortico-cortical visual networks in humans. *Curr. Opin. Behav. Sci.* **56**, 101359 (2024).

26. D. Hebb, *The Organisation of Behaviour* (John Wiley and Sons, 1949).

27. G. Bi, M. Poo, Synaptic modification by correlated activity: Hebb's postulate revisited. *Annu. Rev. Neurosci.* **24**, 134–166 (2001).

28. N. Caporale, Y. Dan, Spike timing-dependent plasticity: A Hebbian learning rule. *Annu. Rev. Neurosci.* **31**, 25–46 (2008).

29. F. Fiori, E. Chiappini, A. Avenanti, Enhanced action performance following TMS manipulation of associative plasticity in ventral premotor-motor pathway. *Neuroimage* **183**, 847–858 (2018).

30. S. Turrini, F. Fiori, N. Bevacqua, C. Saracini, B. Lucero, M. Candidi, A. Avenanti, Spike-timing-dependent plasticity induction reveals dissociable supplementary– and premotor–

motor pathways to automatic imitation. *Proc. Natl. Acad. Sci. U.S.A.* **121**, e2404925121 (2024).

31. J. Silvanto, N. Muggleton, V. Walsh, State-dependency in brain stimulation studies of perception and cognition. *Trends Cogn. Sci.* **12**, 447–454 (2008).

32. J. Silvanto, Z. Cattaneo, Common framework for "virtual lesion" and state-dependent TMS: The facilitatory/suppressive range model of online TMS effects on behavior. *Brain Cogn.* **119**, 32–38 (2017).

33. E. Chiappini, J. Silvanto, P. Hibbard, A. Avenanti, V. Romei, Strengthening functionally specific neural pathways with transcranial brain stimulation. *Curr. Biol.* **28**, R735–R736 (2018).

34. P. O. Jacquet, A. Avenanti, Perturbing the action observation network during perception and categorization of actions' goals and grips: State-dependency and virtual lesion TMS effects. *Cereb. Cortex* **25**, 598–608 (2015).

35. S. Turrini, F. Fiori, E. Chiappini, B. Lucero, E. Santarnecchi, A. Avenanti, Cortico-cortical paired associative stimulation (ccPAS) over premotor-motor areas affects local circuitries in the human motor cortex via Hebbian plasticity. *Neuroimage* **271**, 120027 (2023).

36. E. R. Buch, V. M. Johnen, N. Nelissen, J. O'Shea, M. F. S. Rushworth, Noninvasive associative plasticity induction in a corticocortical pathway of the human brain. *J. Neurosci.* **31**, 17669–17679 (2011).

37. M. Davare, R. Lemon, E. Olivier, Selective modulation of interactions between ventral premotor cortex and primary motor cortex during precision grasping in humans. *J. Physiol.* **586**, 2735–2742 (2008).

38. M. Davare, K. Montague, E. Olivier, J. C. Rothwell, R. N. Lemon, Ventral premotor to primary motor cortical interactions during object-driven grasp in humans. *Cortex* **45**, 1050–1057 (2009).

39. E. Chiappini, S. Turrini, F. Fiori, A. Tessari, M. G. Benassi, G. di Pellegrino, A. Avenanti, You are as old as the connectivity you keep: Distinct neurophysiological mechanisms underlying age-related changes in hand dexterity and strength. *Arch. Med. Res.* **56**, 103031 (2025).

40. H. Markram, W. Gerstner, P. J. Sjöström, A history of spike-timing-dependent plasticity. *Front. Synaptic Neurosci.* **3**, 4 (2011).

41. H. Markram, J. Lübke, M. Frotscher, B. Sakmann, Regulation of synaptic efficacy by coincidence of postsynaptic APs and EPSPs. *Science* **275**, 213–215 (1997).

42. S. Turrini, F. Fiori, E. Chiappini, V. Romei, E. Santarnecchi, A. Avenanti, Gradual enhancement of corticomotor excitability during cortico-cortical paired associative stimulation. *Sci. Rep.* **12**, 14670 (2022).

43. A. Casarotto, E. Dolfini, P. Cardellicchio, L. Fadiga, A. D'Ausilio, G. Koch, Mechanisms of Hebbian-like plasticity in the ventral premotor - primary motor network. *J. Physiol.* **601**, 211–226 (2023).

44. E. Chiappini, S. Turrini, M. Zanon, M. Marangon, S. Borgomaneri, A. Avenanti, Driving Hebbian plasticity over ventral premotor-motor projections transiently enhances motor resonance. *Brain Stimul.* **17**, 211–220 (2024).

45. V. M. Johnen, F.-X. Neubert, E. R. Buch, L. Verhagen, J. X. O'Reilly, R. B. Mars, M. F. S. Rushworth, Causal manipulation of functional connectivity in a specific neural pathway during behaviour and at rest. *eLife* **4**, e04585 (2015).

46. M. N. Thabit, Y. Ueki, S. Koganemaru, G. Fawi, H. Fukuyama, T. Mima, Movement-related cortical stimulation can induce human motor plasticity. *J. Neurosci.* **30**, 11529–11536 (2010).

47. T. Kujirai, M. D. Caramia, J. C. Rothwell, B. L. Day, P. D. Thompson, A. Ferbert, S. Wroe, P. Asselman, C. D. Marsden, Corticocortical inhibition in human motor cortex. *J. Physiol.* **471**, 501–519 (1993).

48. U. Ziemann, J. C. Rothwell, M. C. Ridding, Interaction between intracortical inhibition and facilitation in human motor cortex. *J. Physiol.* **496**, 873–881 (1996).

49. W. Paulus, J. Classen, L. G. Cohen, C. H. Large, V. Di Lazzaro, M. Nitsche, A. Pascual-Leone, F. Rosenow, J. C. Rothwell, U. Ziemann, State of the art: Pharmacologic effects on cortical excitability measures tested by transcranial magnetic stimulation. *Brain Stimul.* **1**, 151–163 (2008).

50. S. Turrini, N. Bevacqua, A. Cataneo, E. Chiappini, F. Fiori, M. Candidi, A. Avenanti, Transcranial cortico-cortical paired associative stimulation (ccPAS) over ventral premotor-motor pathways enhances action performance and corticomotor excitability in young adults more than in elderly adults. *Front. Aging Neurosci.* **15**, 1119508 (2023).

51. M. J. Hautus, N. A. Macmillan, C. D. Creelman, *Detection Theory: A User's Guide* (Routledge, ed. 3, 2021); https://doi.org/10.4324/9781003203636.

52. M. Candidi, B. M. C. Stienen, S. M. Aglioti, B. De Gelder, Event-related repetitive transcranial magnetic stimulation of posterior superior temporal sulcus improves the detection of threatening postural changes in human bodies. *J. Neurosci.* **31**, 17547–17554 (2011).

53. R. P. Dum, P. L. Strick, Spinal cord terminations of the medial wall motor areas in macaque monkeys. *J. Neurosci.* **16**, 6513–6525 (1996).

54. S. Ghosh, R. Porter, Corticocortical synaptic influences on morphologically identified pyramidal neurones in the motor cortex of the monkey. *J. Physiol.* **400**, 617–629 (1988).

55. G. Prabhu, H. Shimazu, G. Cerri, T. Brochier, R. L. Spinks, M. A. Maier, R. N. Lemon, Modulation of primary motor cortex outputs from ventral premotor cortex during visually guided grasp in the macaque monkey. *J. Physiol.* **587**, 1057–1069 (2009).

56. H. Tokuno, A. Nambu, Organization of nonprimary motor cortical inputs on pyramidal and nonpyramidal tract neurons of primary motor cortex: An electrophysiological study in the macaque monkey. *Cereb. Cortex* **10**, 58–68 (2000).

57. M. Davare, J. C. Rothwell, R. N. Lemon, Causal connectivity between the human anterior intraparietal area and premotor cortex during grasp. *Curr. Biol.* **20**, 176–181 (2010).

58. Y. H. Sohn, M. Hallett, Disturbed surround inhibition in focal hand dystonia. *Ann. Neurol.* **56**, 595–599 (2004).

59. J. Trajkovic, V. Romei, M. F. S. Rushworth, A. Sel, Changing connectivity between premotor and motor cortex changes inter-areal communication in the human brain. *Prog. Neurobiol.* **228**, 102487 (2023).

60. A. Sel, L. Verhagen, K. Angerer, R. David, M. C. Klein-Flügge, M. F. S. Rushworth, Increasing and decreasing interregional brain coupling increases and decreases oscillatory activity in the human brain. *Proc. Natl. Acad. Sci. U.S.A.* **118**, e2100652118 (2021).

61. K. Stefan, E. Kunesch, L. G. Cohen, R. Benecke, J. Classen, Induction of plasticity in the human motor cortex by paired associative stimulation. *Brain* **123**, 572–584 (2000).

62. S. Turrini, N. Bevacqua, A. Cataneo, E. Chiappini, F. Fiori, S. Battaglia, V. Romei, A. Avenanti, Neurophysiological markers of premotor-motor network plasticity predict motor performance in young and older adults. *Biomedicine* **11**, 1464 (2023).

63. M. Bologna, L. Rocchi, G. Paparella, A. Nardella, P. Li Voti, A. Conte, M. Kojovic, J. C. Rothwell, A. Berardelli, Reversal of practice-related effects on corticospinal excitability has no immediate effect on behavioral outcome. *Brain Stimul.* **8**, 603–612 (2015).

64. J. Classen, J. Liepert, S. P. Wise, M. Hallett, L. G. Cohen, Rapid plasticity of human cortical movement representation induced by practice. *J. Neurophysiol.* **79**, 1117–1123 (1998).

65. J.-F. Lepage, O. Morin-Moncet, V. Beaulé, L. de Beaumont, F. Champoux, H. Théoret, Occlusion of LTP-like plasticity in human primary motor cortex by action observation. *PLOS ONE* **7**, e38754 (2012).

66. U. Ziemann, R. Chen, L. G. Cohen, M. Hallett, Dextromethorphan decreases the excitability of the human motor cortex. *Neurology* **51**, 1320–1324 (1998).

67. U. Ziemann, TMS and drugs. *Clin. Neurophysiol.* **115**, 1717–1729 (2004).

68. A. M. Vallence, M. C. Ridding, Non-invasive induction of plasticity in the human cortex: Uses and limitations. *Cortex* **58**, 261–271 (2014).

69. W. Muellbacher, U. Ziemann, J. Wissel, N. Dang, M. Kofler, S. Facchini, B. Boroojerdi, W. Poewe, M. Hallet, Early consolidation in human primary motor cortex. *Nature* **415**, 640–644 (2002).

70. E. M. Robertson, D. Z. Press, A. Pascual-Leone, Off-line learning and the primary motor cortex. *J. Neurosci.* **25**, 6372–6378 (2005).

71. R. Chen, D. Cros, A. Curra, V. D. Lazzaro, J.-P. Lefaucheur, M. R. Magistris, K. Mills, K. M. Rösler, W. J. Triggs, Y. Ugawa, U. Ziemann, The clinical diagnostic utility of transcranial magnetic stimulation: Report of an IFCN committee. *Clin. Neurophysiol.* **119**, 504–532 (2008).

72. U. Amadi, C. Allman, H. Johansen-Berg, C. J. Stagg, The homeostatic interaction between anodal transcranial direct current stimulation and motor learning in humans is related to GABAA activity. *Brain Stimul.* **8**, 898–905 (2015).

73. A. Floyer-Lea, M. Wylezinska, T. Kincses, P. M. Matthews, Rapid modulation of GABA concentration in human sensorimotor cortex during motor learning. *J. Neurophysiol.* **95**, 1639–1644 (2006).

74. U. Ziemann, W. Muellbacher, M. Hallett, L. G. Cohen, Modulation of practice-dependent plasticity in human motor cortex. *Brain* **124**, 1171–1181 (2001).

75. M. V. Sale, M. C. Ridding, M. A. Nordstrom, Factors influencing the magnitude and reproducibility of corticomotor excitability changes induced by paired associative stimulation. *Exp. Brain Res.* **181**, 615–626 (2007).

76. U. Castiello, The neuroscience of grasping. *Nat. Rev. Neurosci.* **6**, 726–736 (2005).

77. M. J. Grol, J. Majdandzic, K. E. Stephan, L. Verhagen, H. C. Dijkerman, H. Bekkering, F. A. J. Verstraten, I. Toni, Parieto-frontal connectivity during visually guided grasping. *J. Neurosci.* **27**, 11877–11887 (2007).

78. S. Rossi, A. Antal, S. Bestmann, M. Bikson, C. Brewer, J. Brockmöller, L. L. Carpenter, M. Cincotta, R. Chen, J. D. Daskalakis, V. Di Lazzaro, M. D. Fox, M. S. George, D. Gilbert, V. K. Kimiskidis, G. Koch, R. J. Ilmoniemi, J. P. Lefaucheur, L. Leocani, S. H. Lisanby, C. Miniussi, F. Padberg, A. Pascual-Leone, W. Paulus, A. V. Peterchev, A. Quartarone, A. Rotenberg, J. Rothwell, P. M. Rossini, E. Santarnecchi, M. M. Shafi, H. R. Siebner, Y. Ugawa, E. M. Wassermann, A. Zangen, U. Ziemann, M. Hallett, Safety and recommendations for TMS use in healthy subjects and patient populations, with updates on training, ethical and regulatory issues: Expert Guidelines. *Clin. Neurophysiol.* **132**, 269–306 (2021).

79. World Medical Association, World Medical Association Declaration of Helsinki: Ethical principles for medical research involving human subjects. *JAMA* **310**, 2191–2194 (2013).

80. J. A. Camprodon, J. Martínez-Raga, M. Alonso-Alonso, M.-C. Shih, A. Pascual-Leone, One session of high frequency repetitive transcranial magnetic stimulation (rTMS) to the right prefrontal cortex transiently reduces cocaine craving. *Drug Alcohol Depend.* **86**, 91–94 (2007).

81. F. Jetté, I. Côté, H. B. Meziane, C. Mercier, Effect of single-session repetitive transcranial magnetic stimulation applied over the hand versus leg motor area on pain after spinal cord injury. *Neurorehabil. Neural Repair* **27**, 636–643 (2013).

82. G. Koch, V. Ponzo, F. D. Lorenzo, C. Caltagirone, D. Veniero, Hebbian and anti-Hebbian spike-timing-dependent plasticity of human cortico-cortical connections. *J. Neurosci.* **33**, 9725–9733 (2013).

83. D. Veniero, V. Ponzo, G. Koch, Paired associative stimulation enforces the communication between interconnected areas. *J. Neurosci.* **33**, 13773–13783 (2013).

84. T. Kammer, S. Beck, A. Thielscher, U. Laubis-Hermann, H. Topka, Motor threshold in humans: A transcranial magnetic stimulation study comparing different pulse waveforms, current directions and stimulator types. *Clin. Neurophysiol.* **112**, 250–258 (2001).

85. P. M. Rossini, D. Burke, R. Chen, L. G. Cohen, Z. Daskalakis, R. Di Iorio, V. Di Lazzaro, F. Ferreri, P. B. Fitzgerald, M. S. George, M. Hallett, J. P. Lefaucheur, B. Langguth, H. Matsumoto, C. Miniussi, M. A. Nitsche, A. Pascual-Leone, W. Paulus, S. Rossi, J. C. Rothwell, H. R. Siebner, Y. Ugawa, V. Walsh, U. Ziemann, Non-invasive electrical and magnetic stimulation of the brain, spinal cord, roots and peripheral nerves: Basic principles and procedures for routine clinical and research application. An updated report from an I.F.C.N. Committee. *Clin. Neurophysiol.* **126**, 1071–1107 (2015).

86. E. Chiappini, S. Borgomaneri, M. Marangon, S. Turrini, V. Romei, A. Avenanti, Driving associative plasticity in premotor-motor connections through a novel paired associative stimulation based on long-latency cortico-cortical interactions. *Brain Stimul.* **13**, 1461–1463 (2020).

87. M. Dafotakis, R. Sparing, S. B. Eickhoff, G. R. Fink, D. A. Nowak, On the role of the ventral premotor cortex and anterior intraparietal area for predictive and reactive scaling of grip force. *Brain Res.* **1228**, 73–80 (2008).

88. M. A. Mayka, D. M. Corcos, S. E. V. D. E. Leurgans, D. E. Vaillancourt, Three-dimensional locations and boundaries of motor and premotor cortices as defined by functional brain imaging: A metaanalysis. *Neuroimage* **31**, 1453–1474 (2006).

89. H. Devanne, B. A. Lavoie, C. Capaday, Input-output properties and gain changes in the human corticospinal pathway. *Exp. Brain Res.* **114**, 229–238 (1997).

90. J. Cohen, *Statistical Power Analysis for the Behavioral Sciences* (Lawrence Earlbaum Associates, 1988).

91. J. Pallant, *SPSS Survival Manual: A Step by Step Guide to Data Analysis using IBM SPSS* (McGraw-Hill Education, 2020); https://doi.org/10.4324/9781003117452.

92. C. Catmur, C. Heyes, Time course analyses confirm independence of imitative and spatial compatibility. *J. Exp. Psychol. Hum. Percept. Perform.* **37**, 409–421 (2011).
